# Algorithmic Explorations of Unimolecular and Bimolecular Reaction Spaces**

**DOI:** 10.1002/anie.202210693

**Published:** 2022-10-17

**Authors:** Qiyuan Zhao, Brett M. Savoie

**Affiliations:** ^1^ Davidson School of Chemical Engineering Purdue University West Lafayette IN 47906 USA

**Keywords:** Automated Reaction Prediction, Conformational Sampling, Reaction Mechanisms, Reaction Networks, Transition States

## Abstract

Algorithmic reaction exploration based on transition state searches has already made inroads into many niche applications, but its potential as a general‐purpose tool is still largely unrealized. Computational cost and the absence of benchmark problems involving larger molecules remain obstacles to further progress. Here an ultra‐low cost exploration algorithm is implemented and used to explore the reactivity of unimolecular and bimolecular reactants, comprising a total of 581 reactions involving 51 distinct reactants. The algorithm discovers all established reaction pathways, where such comparisons are possible, while also revealing a much richer reactivity landscape, including lower barrier reaction pathways and a strong dependence of reaction conformation in the apparent barriers of the reported reactions. The diversity of these benchmarks illustrate that reaction exploration algorithms are approaching general‐purpose capability.

## Graphical abstract

An ultra‐low cost reaction exploration algorithm (Yet Another Reaction Program, v2.0) is used to predict reaction outcomes and characterize multiple unimolecular and bimolecular reaction networks. The diversity of these benchmarks illustrate that reaction exploration algorithms are approaching general‐purpose capability.

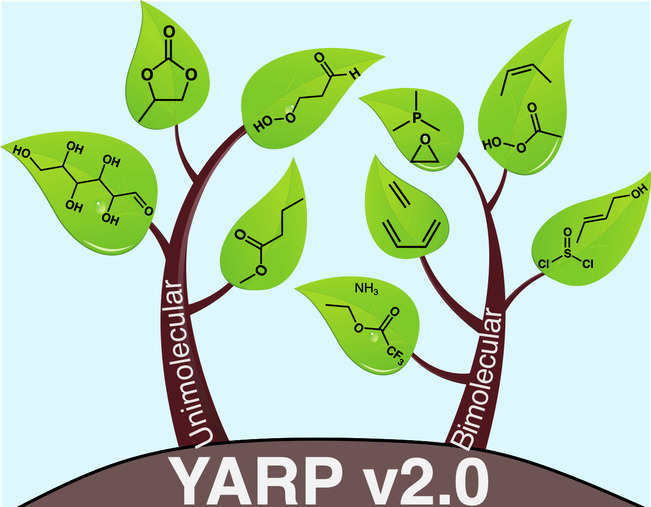

## Introduction

Automated reaction prediction methods have been developed for decades[^1^, ^2^, ^3^, ^4^] and applied to various of research fields, including combustion chemistry,[^5^, ^6^] low‐temperature atmospheric chemistry,[^7^, ^8^, ^9^] and biomass conversion.[^10^, ^11^] Although many traditional network exploration algorithms depend on tabulated reaction rules, recent methods development has been focused on searches based on localizing transition states (TSs) on the atomic potential energy surface (PES).[^12^, ^13^, ^14^, ^15^, ^16^, ^17^, ^18^, ^19^] A major advantage of this paradigm is that reaction rules do not necessarily need to be known in advance, which creates the opportunity for discovering new reactions and applying these methods in predictive and exploratory scenarios. Nevertheless, high computational costs and inconsistent reaction exploration remain serious impediments for the black‐box use of contemporary search algorithms. Moreover, the absence of benchmark problems for reaction exploration makes it difficult to measure progress and identify the relative advantage of various methods.

The goal of a quantum chemistry based exploration algorithm is to discover all kinetically and thermodynamically relevant reactions associated with a given set of reactants and reaction conditions. By considering discovered products as potential further reactants, these algorithms can also be applied recursively for elucidating multi‐step reaction networks. The major distinctions among the various methods that have been employed to date consist in how transition states are searched for and how the PES is approximated. For example, the artificial force induced reaction (AFIR) family of methods that has been in development for over a decade by Maeda et al. consist of using a heuristic to force reactive atoms together to find low‐barrier transition states.^[14]^ AFIR is representative of so‐called “single‐ended” methods that only use information of the reactant side of the PES when searching for TSs. In contrast, the Z‐Struct suite of methods developed by Zimmerman is representative of double‐ended graph based methods for finding reactions.^[12]^ Double‐ended methods use a product enumeration algorithm, typically based on generic graph‐based reaction rules or user‐defined templates, to identify nearby regions of the PES that are possibly connected by a single TS to the reactant.^[20]^ The advantage of double‐ended methods are that the TS searches are psuedo‐one dimensional and, thus, much faster. Neither of the foregoing examples are dependent on a particular representation of the PES. However, several recent algorithms are distinguished by their use of machine‐learning (ML) based approximations of the PES that nominally allow better exploration of the reaction space. For example, Liu et al. have developed the stochastic surface walking method (SSWM) that trains a system specific neural‐network potential as a preliminary step, then uses this as a PES surrogate for identifying reaction pathways.^[11]^ Mixtures of these strategies are used by many other algorithms. For example, the nanoreactor approach uses an inexpensive model chemistry to run molecular dynamics under conditions that promote reactions, then refines the discovered reactions at higher levels using double‐ended searches.[^21^, ^22^] Habershon et al. have developed a method that combines the idea of graph‐based reaction enumeration with discreet optimization, this yields multistep reaction pathways in terms of graphical operations that are subsequently structurally refined.^[17]^

Unfortunately, the proliferation of such algorithms has not been paralleled by the availability of shared benchmark problems and performance statistics. In developing such benchmarks, two aspects of these methods are critical for assessing performance. The first is whether a given method correctly identifies all relevant products and their low‐barrier TSs under given reaction conditions. Any given method will find an array of reaction channels, but without a direct comparison against either detailed experimental data or other algorithms applied to the same problem the actual relevance of discovered pathways is unknown. To the best of our knowledge, a recent study by Grambow et al.^[23]^ (combined with revised AFIR results later published by Maeda et al.^[24]^) represents the only head‐to‐head comparison of this kind, albeit only for three distinct families of methods and a single unimolecular degradation problem. When it comes to assessing accuracy there are also the related questions of whether the lowest barrier transition state for a given reaction is discovered (e.g., a change of conformation can qualitatively modulate barriers and reaction mechanisms),[^25^, ^26^] whether the rank ordering of various channels is correct (e.g., a method with inaccurate absolute barriers that still reliably predicts the major product could still be useful), the level of theory of the final PES (e.g., choice of functional or use of a solvent model), whether the transition states are actually validated (e.g., using pathway sampling or an intrinsic reaction coordinate [IRC] calculation), and whether multi‐step reactions yielding identical products are compared against single‐step reaction channels. The second critical aspect for assessing performance is the computational cost of running the algorithm. Existing algorithms can wildly differ in terms of cost and scaling, and reporting is haphazard. For example, the poor scaling with respect to system size of many algorithms is obfuscated by only applying them to small systems involving a few heavy atoms. Ideally, a cost breakdown of the various steps of the algorithm expressed in a resource independent measure should be used. For example, we have adopted the number of high‐level gradient calls per validated reaction channel as a useful metric, since high‐level gradient calls are the most expensive step of our algorithm. Here, “validated” means that the TS has been confirmed to correspond to the putative reaction diagram. This accounts for the fact that localizing unintended transitions states is a common failure mechanism of many algorithms.[^23^, ^27^] This metric is also resource independent in a way that walltime is not, since parallelization details and hardware affect the cost of gradient calls. An extension of such a metric might be number of gradient calls per discovered low barrier reaction. This would reflect the difference between an algorithm that wastes time characterizing irrelevant reactions and one that economizes the selection of reactions to characterize. Of course, such a metric could only be meaningfully applied to a benchmark where the low‐barrier TSs were already established and would need to be compared with the rate of missed reaction pathways. Many additional ideas are possible, and adaptations to fit the most costly steps of other algorithms will be necessary, but to pursue any of these ideas requires the availability some standardized reaction exploration problems for the community to engage with.

To address these gaps, a new series of benchmarks are reported that comprise 540 distinct reactions from across organic chemistry. Among these are four distinct unimolecular decomposition networks and 122 single‐step bimolecular reactions involving C,H,O,N,S, and P. The data generated as part of these benchmarks include competing TSs for each set of reactants, distinct TS conformers for each reaction, intrinsic reaction coordinate (IRC) characterizations, and general measures of computational cost characterized using the updated version of the Yet Another Reaction Program (YARP) algorithms. Comparing performance in these distinct benchmarks reveals several avenues for improving reaction discovery algorithms, including screening reaction candidates with low‐level methods, using composite double‐ended and single‐ended searches to discover reactions, and using machine learning models to prioritize reaction channels for characterization.

## Results and Discussion

### YARP (v2.0)

The reaction explorations are carried out by YARP, which is a python package developed by our group for characterizing reaction networks.^[27]^ YARP uses generic graph‐based enumeration to generate candidate products that are then characterized by a combination of double‐ended transition state (TS) searches and TS refinement techniques. Among the distinct features of YARP are the handling of conformers for conditioning the TS search and the use of multiple levels of theory to screen and refine TSs. Compared with contemporary methods, YARP dramatically reduces computational costs for characterizing organic reactions in the gas‐phase[^26^, ^27^] and at catalytic surfaces.^[28]^ Nevertheless, as described in the original YARP publication, we envisioned several opportunities to further improve the method. These have now been incorporated into a second version of the software (YARP v2.0) that is used here for benchmark development. These improvements consist of four major components. First, TAFFI Component Increment Theory (TCIT)[^29^, ^30^] is now used to predict the heats of reaction ($\Delta H_{r}^{\circ }$ ) in an on‐the‐fly manner to down‐select pathways for more costly TS searches. Second, reaction conformational sampling (RCS)^[26]^ is applied to provide multiple high quality reaction conformations for each TS search. Third, low‐level methods are applied to the entire TS characterization workflow, including the double‐ended search, Berny TS optimization, and IRC calculations. Fourth, the data from unsuccessful TS searches are mined for potentially relevant reaction pathways.

**Figure 1:**
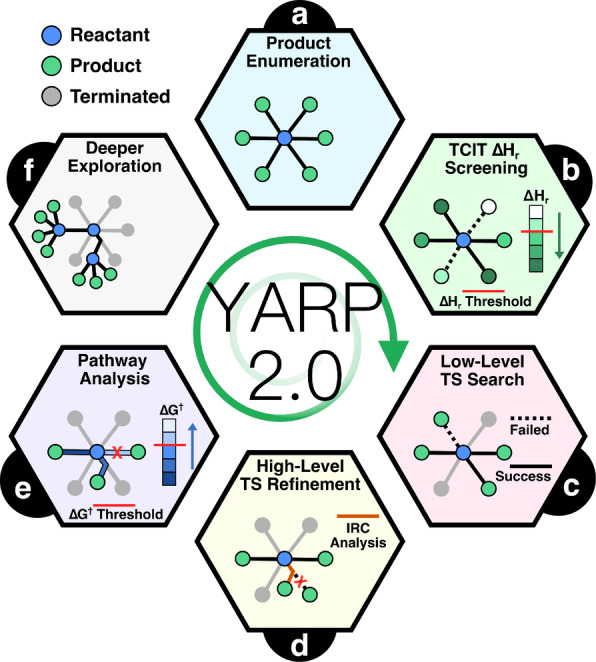
Overview of the reaction exploration methodology (YARP v2.0) used in this work. a) Potential products are enumerated using graph‐based elementary reaction steps. b) The heats of reaction ($\Delta H_{r}^{⊖}$ ) are computed by TCIT and used to down‐select reactions for subsequent characterizations. c) Reaction pathways are characterized at a low level of theory using reaction conformational sampling, double‐ended searches, TS optimization, and IRC calculations. Duplicated reaction channels and optional filtering criteria are applied at this step based on the low‐level results. d) Down‐selected TSs undergo TS refinement and IRC calculations at a higher level of theory. e) Products are added to the reactant pool based on kinetic relevance. f) A new round of ERS enumeration is initiated for the expanded list of reactants.

With these additions, the updated workflow of YARP v2.0 consists of five components for automatically exploring reaction networks: graph‐based product enumeration using elementary reaction steps (ERSs), pre‐pruning products based on the enthalpy changes ($\Delta H_{r}^{\circ }$ ), low‐level reaction pathway characterization and down‐selection, high‐level transition state (TS) refinement, IRC calculation, and reaction relevance analysis (Figure 1a–e). These five steps are recursively applied to the initial reactant(s) and the intermediates discovered during the exploration to characterize the network (Figure 1f). For the basic concept of ERSs and reaction characterization methodology, we direct readers to our previous publication.^[27]^ A detailed description of the new aspects of YARP v2.0 and the settings adopted for each system are provided in the Supporting Information (Section 1). For additional benchmarks, including a comparison of the activation energies calculated with different functionals and CCSD(T)‐F12,[^31^, ^32^] we direct readers to the Supporting Information.

### Unimolecular Degradation Networks

The first set of benchmarks correspond to the unimolecular thermal degradation networks of *γ*‐ketohydroperoxide (KHP), propylene carbonate (PC), and methyl butanoate (MB), which are important molecules in combustion,[^33^, ^34^] battery electrolytes,^[35]^ and biodiesel.^[10]^ These molecules are large enough (6–7 heavy atoms) to challenge many algorithms that poorly scale with size. Specific reaction channels for PC and MB have been previously studied using computational methods, however these reactants have not been studied using general exploration algorithms. KHP was the subject of the only other benchmark study on reaction exploration algorithms. These networks were derived under the assumption that there are no external reactants and that the reactions between intermediates can be neglected. Each network was built up in two iterations of reaction exploration. Namely, product enumeration, filtering steps, and TS localization were performed as described in the methods section (Supporting Information section 1) for each reactant. Then the discovered products were treated like new reactants and the YARP cycle (Figure 1) was repeated. Only neutral closed‐shell products were treated as candidates for the second exploration. An overview of each network and the comparison between them illustrate the diversity of kinetically competitive reaction channels accessible via relatively small reacting systems.

**Figure 2:**
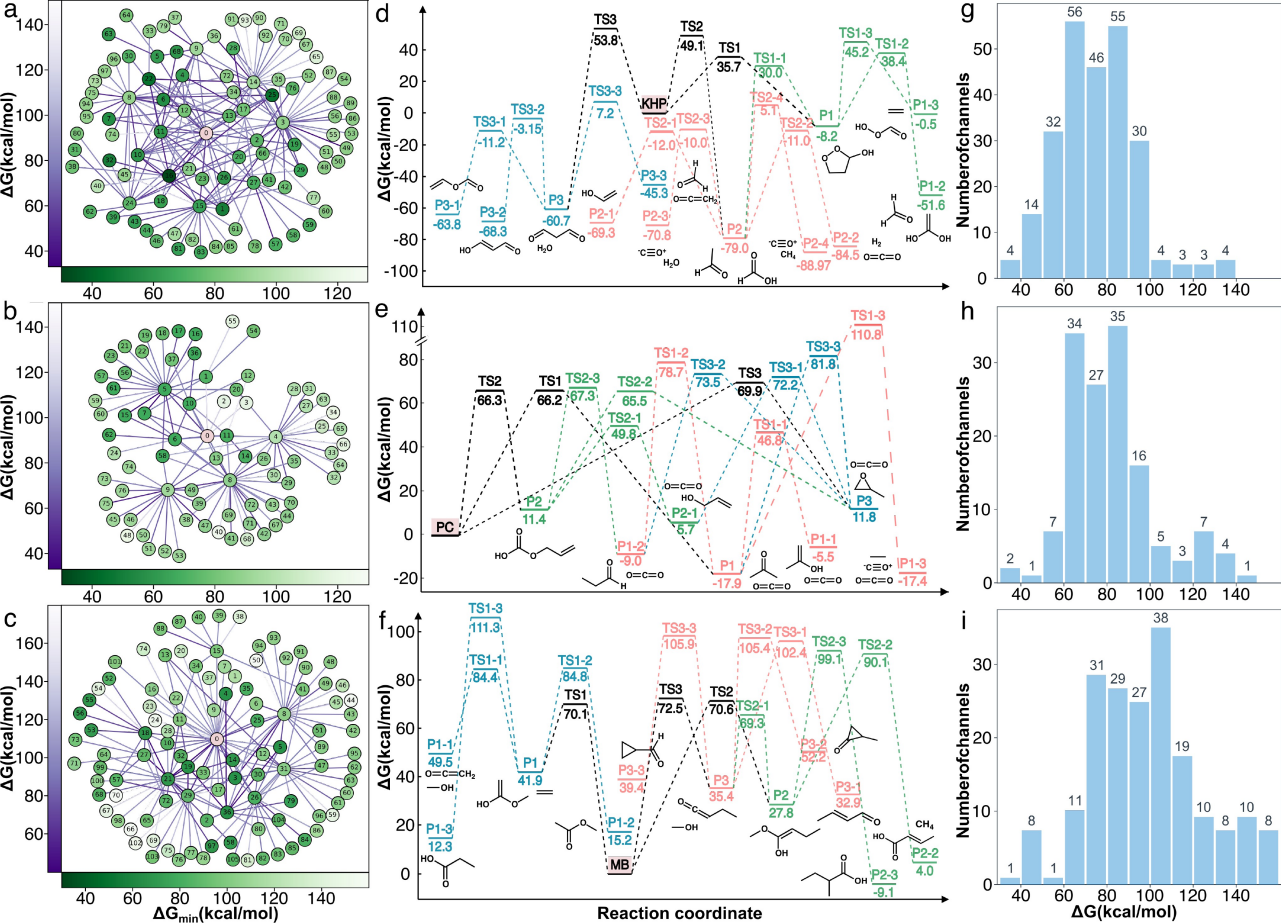
Unimolecular decomposition studies of KHP, PC and MB. a)–c) Degradation networks depicting activation barriers (computed at M052X‐D3/def2‐SVP level of theory) for rate‐limiting steps as the node colors and individual activation barriers as the edge colors. d)–f) The most kinetically favorable degradation pathways for each species defined as the pathway exhibiting the lowest maximum barrier along its pathway. Energies are computed at *ω*B97XD/def2‐TZVP level of theory. Different line colors represent different starting points (i.e. reactants and intermediates). g)–i) Activation energy distributions for all reactions from each network. The panels on the first, second and third row correspond to the results for the KHP, PC and MB degradation networks, respectively.

The degradation network of KHP consists of 98 distinct reactants and products (nodes) connected by 205 distinct reactions (edges, Figure 2a). Starting from KHP, 20 single‐step reactions (13 intended and 7 unintended) were discovered that produced 27 products. Among those products, 7 small molecules (containing less than three non‐hydrogen atoms, like water) and 1 diradical species (propan‐1,1‐diyl, [CH]CC) were excluded from further exploration, while the other products underwent a second step of unimolecular reaction exploration. Based on their overall minimum activation barriers to formation, formaldehyde (node 1), prop‐2‐enal (6), hydrogen peroxide (7), 1,2‐dioxolan‐3‐ol (16), acetaldehyde (22), formic acid (25), and ethene‐1,1‐diol (32) are the most kinetically favorable products, respectively. More detailed reaction pathways are provided in an energy profile diagram (Figure 2d), from which we observe that KHP is a relatively unstable compound on the PES and all of the kinetically favored reactions are exothermic. More importantly, the lowest barrier unimolecular decomposition pathway discovered by YARP is the Korcek mechanism, in which a five‐membered cyclic peroxide (1,2‐dioxolan‐3‐ol) first forms, and then decomposes into acetaldehyde and formic acid. This pathway matches the mechanism proposed by Jalan, A. et al.^[36]^ In addition, kinetically competitive pathways were also discovered for producing formaldehyde (P3‐3), ethene (P1‐3), carbon monoxide (P2‐3) and carbon dioxide (P2‐2).

The degradation network of PC consists of 77 distinct reactants and products (nodes) connected by 119 distinct reactions (edges, Figure 2b). Starting from PC, 9 single‐step reactions (7 intended and 2 unintended) were identified that lead to 11 products. Among those products, methane (node 2) and carbon dioxide (7) were excluded from further exploration while the nine other products underwent a second step of unimolecular reaction exploration. Four of the initial products [2‐oxoethyl acetate (node 4), prop‐2‐enyl hydrogen carbonate (5), 2‐oxopropyl formate (8) and 1‐oxopropan‐2‐yl formate (9)] are linear isomers of PC and yield the vast majority of intended second layer reaction channels. Among these linear isomers, prop‐2‐enyl hydrogen carbonate (5) is the most kinetically favorable and dominates the second‐layer reactions. Carbon dioxide (node 7) serves as a common product during the degradation, while 2‐methyloxirane (6), propanal (10), propan‐2‐one (11), prop‐2‐en‐1‐ol (15), prop‐1‐en‐1‐ol (36), formaldehyde (58), and ethene (61) are all likely to occur based on the $\Delta G^{†}$ of their respective rate‐limiting steps.

The decomposition networks of PC and KHP exhibit several marked differences. First, the range of $\Delta H_{r}^{⊖}$ of kinetically favorable reactions is relatively narrow and close to zero on average. This reflects the much more stable nature of PC with respect to unimolecular decomposition compared with KHP. Secondly, the topology of the PC network is dominated by traversal through the four linear isomers of PC, whereas the KHP network is much more densely connected. This is qualitatively consistent with the much higher exothermicity of the KHP network. Finally, the interconversion of products occupies a significant proportion of the overall PC decomposition network (Figure 2e). For instance, propan‐2‐one (P1), 2‐methyloxirane (P3), and propanal (P1‐2) are all accessible from PC and prop‐2‐enyl hydrogen carbonate (P2) (The reaction pathway from PC to P1‐2 has a slightly higher barrier that results in its exclusion from Figure 2e) and the difference between the activation energies associated with the interconversion of these species is within 5 kcal mol^−1^.

The degradation network of MB consists of 106 distinct products (nodes) connected by 166 distinct reactions (edges, Figure 2c). Starting from MB, 30 reactions (20 intended and 10 unintended) were identified that lead to 37 products. MB is relatively stable to unimolecular degradation compared with KHP and PC, with only a single exothermic product discovered (Figure 2f) and the $\Delta G^{†}$ distribution shifted to higher values than the previous systems (Figure 2g‐i). The 37 direct products discovered from MB degradation include all closed‐shell products and reactions with similar $\Delta G^{†}$ to those proposed in a previous study of MB.^[10]^ The higher number of first step reactions compared with the previous networks is also consistent with the more conservative $\Delta H_{r}$ threshold used for filtering these reactions. To limit the exploration to kinetically relevant products, a threshold on the activation energy of $\Delta G^{†}>$ 105.9 kcal mol^−1^ (i.e., the barrier of the MB to methyl acetate, node 4, reaction in Figure 2c) was used to down‐select 12 products for subsequent exploration. These products include all of those reported by Akbar Ali, M et al.^[10]^ for MB as a subset. Out of the final set of 105 products, the $\Delta G^{†}$ of the rate‐limiting steps of producing ethene (node 3), methyl acetate (4), ketene (5), methanol (19) and 1‐methoxyethenol (36) are below 70 kcal mol^−1^ (i.e., approximately the lowest activation energy previously reported for reaction intermediates of MB). Notably, a low barrier pathway for forming methyl acetate (node 4) was discovered by YARP. The direct reaction from MB to methyl acetate, as reported by Akbar Ali, M et al.^[10]^ and also discovered by YARP, is unfavorable with a $\Delta G^{†}$ over 100 kcal mol^−1^. However, a two‐step reaction mechanism from MB to 1‐methoxyethenol (node 36) then to methyl acetate occurs via much lower activation energies of 68.2 and 44.2 kcal mol^−1^, respectively. This pathway is compared with other kinetically competitive unimolecular channels, including the overall exothermic reaction to 2‐methylbutyric acid (P2‐3), in the accompanying energy diagram (Figure 2f).

### Performance Statistics for Unimolecular Networks

Four statistics are reported to quantify the efficiency of the automated reaction prediction tasks: success rate, intended rate, total number of gradient calls (GCs) and gradient calls per intended product (GCPI). The success rate is defined as the fraction of products characterized at the high‐level (i.e., DFT in these benchmarks) whose high‐level TS search localized at least one TS. The intended rate is the same as the success rate, except that the high‐level TS has the additional constraint of being intended (i.e., the intended rate is bound from above by the success rate since not all discovered TSs are intended). The total number of high‐level gradient calls has been previously used as a resource independent measure of computational cost for characterizing reactions.[^23^, ^37^] In the case of YARP, the high‐level gradient calls dominate the overall cost of the exploration.^[27]^ In this study, we continued to use this statistic and additionally report the GPCI which is the total number of GCs divided by the total number of intended products. Normalizing by the number of intended products better expresses the efficiency of the search, since a small GC total might trivially be associated with a small number of attempted reactions. We also report the $\Delta G^{†}$ range observed for TSs localized from different reaction conformations. This statistic is somewhat unique to these benchmarks, since conformational sampling is generally not performed for TSs due to the additional computational cost. The distribution of the $\Delta G^{†}$ range across distinct reaction channels is reported to quantify the potential importance of conformational effects in determining the effective barrier height.^[26]^

**Figure 3:**
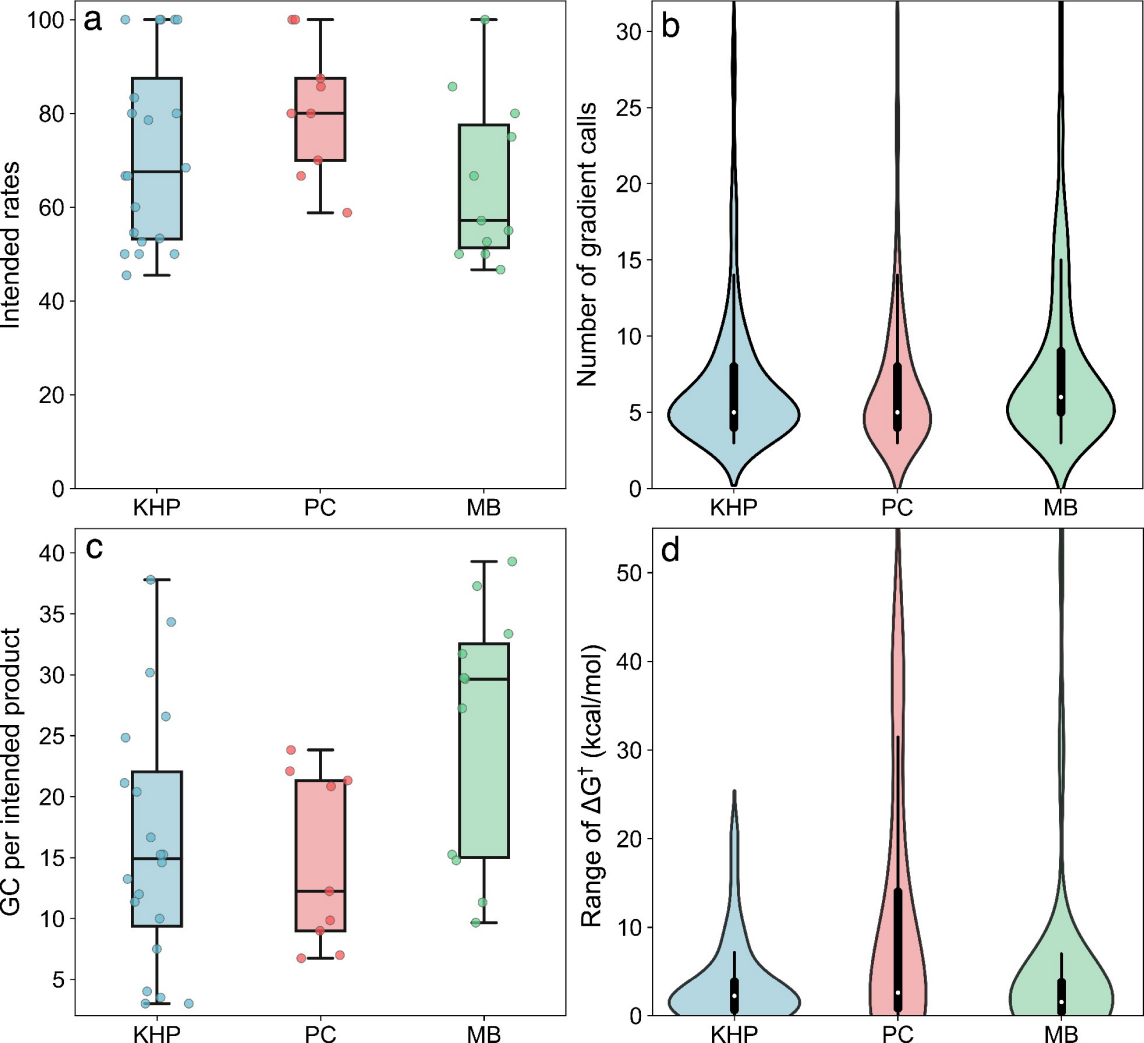
Performance statistics for the unimolecular decomposition studies of KHP, PC, and MB. a) Box and whisker plot of the intended rates for unique reactions involving each reactant. b) The distribution of the number of DFT gradient calls required to localize all TSs (c) Box and whisker plot of the average number of DFT gradient calls required to discover unique intended products for each reactant. Each point is calculated as all GCs associated with reaction exploration for a given reactant divided by the number of distinct intended products. d) The distribution of the range of $\Delta G^{†}$ among different reaction conformations associated with each reaction

The success rate for converging TSs connecting reactants to each attempted product is nearly 100 % in all cases. This is an extremely high value that reflects the efficiency of the prefiltering process in removing unrealistic products and reaction conformations in YARP v2.0 (see Figure S4 for a detailed breakdown of how many products are discarded due to various filtering criteria). YARP v2.0 also achieved extremely high average intended rates of 61.3 %, 77.8 % and 59.3 % for the KHP, PC, and MB explorations, respectively (Figure 3a). For reference, without such filtering, YARP v1.0 achieved success and intended rates of 88.8 % and 54.7 % on the KHP benchmark, and the DFT‐based methods benchmarked by Grambow on KHP exhibited success and intended rates ranging from 23–66 % and 3–19 % respectively. Notably, the intended rate has further increased for YARP v2.0 despite the fact that we are now intentionally retaining unintended channels involving unexpected products, which contributes to inflating the unintended rate.

The total number of DFT GCs required to localize all TSs for the KHP, PC and MB networks was 2440, 1257 and and 2350 (Figure 3b), respectively. For reference, YARP v1.0 required 8364 DFT GCs in an earlier KHP benchmark,^[27]^ and the non‐template based methods benchmarked by Grambow on KHP required between 65420‐756227 GCs.^[23]^ This reflects nearly a 16‐fold efficiency increase per reaction conformer over YARP v1.0, since four reaction conformers were considered for each reaction here, whereas YARP v1.0 only used a single conformer. Even setting aside the fact that it is exploring multiple reaction conformers, YARP v2.0 exhibits a ≈4‐fold and ≈300‐fold decrease in absolute GCs compared with v1.0 and the earlier GSM‐based KHP benchmark,^[23]^ respectively. The number of high‐level gradient calls per reaction is remarkably low, averaging 6.9, 6.9, and 8.1 for the reactions in the KHP, PC, and MB networks, respectively (Figure 3b), illustrating the generally large configurational overlap between the low‐level approximate TS geometries and the high‐level TS geometries. Nevertheless there are several outliers that required a relatively large number of steps. Sometimes this occurs due to a conformational change during the DFT relaxation, in other cases it reflects a breakdown of the GFN2‐xTB model chemistry. This point will be revisited in subsequent work. The average GCPIs for the KHP, PC, and MB networks are 22.5, 18.0, and 27.6, respectively (Figure 3c). These values are approximately three times higher than the average GC per reaction in each network, indicating that a disproportionate amount of time is spent relaxing unintended channels and multiple conformations.

Finally, it is reasonable to ask whether the additional work spent by YARP v2.0 on localizing TSs for distinct reaction conformers has any impact on the resulting barriers. Multiple TSs were converged for 124 out of the 289 intended reactions comprising these networks. For the 165 reactions with only one discovered TS, the other conformers either failed to localize a TS, localized to unintended TSs, or localized to identical TSs. If we calculate the intended rate based on the most highly ranked reaction conformer (rather than all four that were attempted) the intended rates decrease by 54 %, 64 % and 49 % for KHP, PC, and MB, respectively. This illustrates the centrality of conformational sampling for converging even a single intended transition state. Additionally, for the 124 reactions with multiple intended TSs, the effect of the TS conformation on barrier heights can be estimated from the range of $\Delta G^{†}$ across the intended TSs (Figure 3d). The average $\Delta G^{†}$ ranges are 3.7, 10.0 and 4.5 kcal mol^−1^, corresponding to 56, 24, and 44 reactions in the KHP, PC, and MB systems, respectively (the overall average is 5.2 kcal mol^−1^). This suggests that the mean error associated with choosing the wrong reaction conformer can easily exceed the expected accuracy of DFT in predicting $\Delta G^{†}$ , or worse, localize an unintended TS. This illustrates that conformational sampling needs to be taken seriously for establishing useful reaction benchmarks, since performing higher level calculations on a kinetically irrelevant TS conformation is a waste of effort. A more detailed discussion of 11 reactions that exhibit a $\Delta G^{†}$ range larger than 15 kcal mol^−1^ across various conformers is provided in Figure S8.

### Unintended Reaction Channels

**Figure 4:**
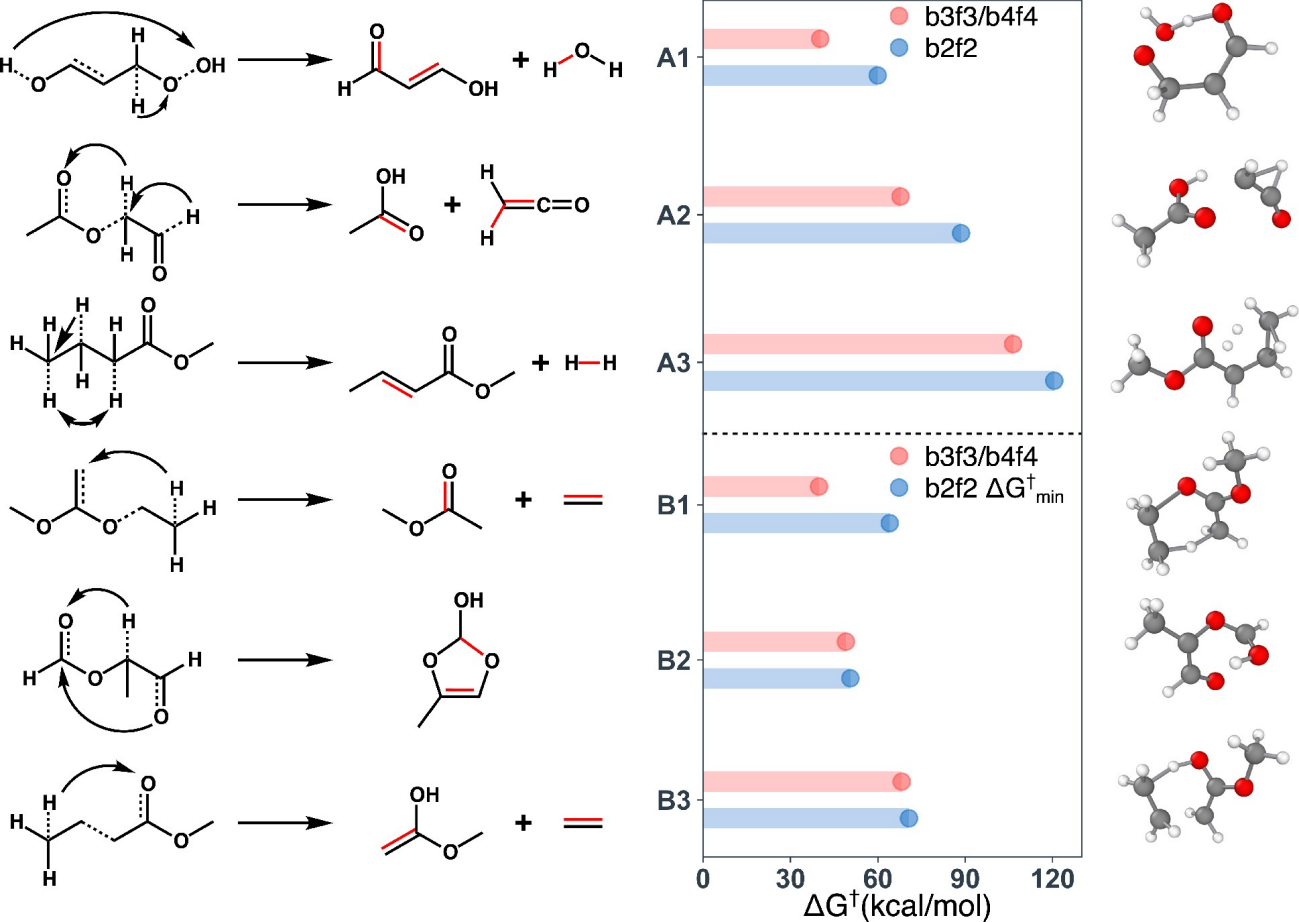
Kinetically relevant unintended reactions discovered during the unimolecular decomposition studies. (left) Reaction mechanisms with broken bonds shown as dotted lines and formed bonds shown in red. (right) a comparison of the activation energies, $\Delta G^{†}$ , for the associated b2f2 reaction and the unintended b3f3/b4f4 reaction. (Class A) These are products where a lower $\Delta G^{†}$ b3f3/b4f4 pathway was discovered compared with the b2f2 pathway to the same product (compared on right). (Class B) These are cases where the unintended b3f3/b4f4 reaction resulted in a product that was not found via alternative b2f2 pathways and that was also lower $\Delta G^{†}$ compared with all other competing b2f2 reactions (compared on right). All $\Delta G^{†}$ are computed at M052X‐D3/def2‐SVP level of theory.

Although b2f2 enumeration was used to initialize all TS searches for these network benchmarks, TSs corresponding to a broad variety of ERSs were still discovered through unintended TSs. This occurs when there is large configurational overlap between a more kinetically favorable pathway and the string of the putative reaction guiding the double‐ended TS search. Ionic and radical products (e.g., homolytic and heterolytic b2f1 reactions yield diradicals and ions, respectively) and also products with a larger number of bond changes (e.g., b4f4 reactions) were discovered through unintended TSs. In many cases, these pathways are just curiosities and are not competitive with the overall lowest barrier reaction for a given set of reactants, but there were six unintended reactions within the benchmarks that qualitatively affect the interpretation of the most kinetically relevant pathways (Figure 4).

In three cases (Figure 4, class A), lower barrier unintended single‐step b3f3 or b4f4 reaction channels were discovered to products that were also found by single‐step intended b2f2 reactions. These unintended reactions all involve TSs with a stable ring structure that make a concerted multi‐bond rearrangement kinetically preferable to the b2f2 pathway. For instance, the TSs with 7‐, 5‐, and 6‐membered ring structures in reactions A1, A2, and A3 reduce $\Delta G^{†}$ by 20, 21 and 14 kcal mol^−1^, respectively, compared with the rate‐limiting step along the b2f2 reaction pathway. Thus, the kinetic relevance of these products would have been grossly underestimated based on the b2f2 pathways. For three other reactions (Figure 4, class B), unintended single‐step b3f3 or b4f4 reactions were discovered for products that no alternative b2f2 pathways were discovered for. These reactions are also lower barrier than all other competing b2f2 reactions. Thus, these are kinetically favored products that would have been missed if the unintended channels had not been retained in the network. Reactions B1 and B3 exhibit a TS for proton transfer involving a 6‐membered ring. This mechanism was also discussed in a previous MB decomposition study.^[10]^ The TS of B2 contains a 7‐membered ring and cyclic product. Notably, all of these cases involve b3f3 or b4f4 reactions and do not produce ions or radicals, which tend to be endothermic and result from high barrier reactions.

Many other kinetically accessible unintended reactions were discovered, albeit not the overall lowest barrier reaction as in the previous cases (all products can be found in the Supporting Information). This demonstrates that the combination of DES and partial single‐ended exploration (via unintended channels involving the reactants) can increase the reaction coverage rate while avoiding the significant increase in computational cost by performing brute‐force b3f3 or even b4f4 enumeration. A more detailed comparison of applying b2f2 and b3f3 ERSs to PC decomposition is provided in Supporting Information section 6 that illustrates including unintended channels can be much more efficient than generally applying higher order ERSs (e.g. b3f3 and b4f4) to increase the reaction coverage.

### One‐step Bimolecular Reaction Exploration

There are many scenarios where reaction exploration algorithms are needed for evaluating competing single‐step reactions, rather than a full network. Moreover, exploration algorithms need to be accurate across diverse reactant chemistries, including bimolecular reactants. To establish benchmarks for these scenarios, we used algorithmic reaction exploration to characterize the competing single‐step reactions of 10 bimolecular reactants curated by Zimmerman,^[38]^ covering the elements C, H, O, N, S, and P and containing up to 9 heavy (non‐hydrogen) atoms. Zimmerman provided transition states for established reaction mechanisms for each reactant that can be compared with the lowest barrier reactions discovered from the set of algorithmically discovered pathways. Attempting to localize TSs for all b2f2 reactions that satisfied the filtering criteria described in the methods section (Supporting Information section 1) resulted in a total of 124 distinct products and 83 distinct intended reactions being discovered.

**Figure 5:**
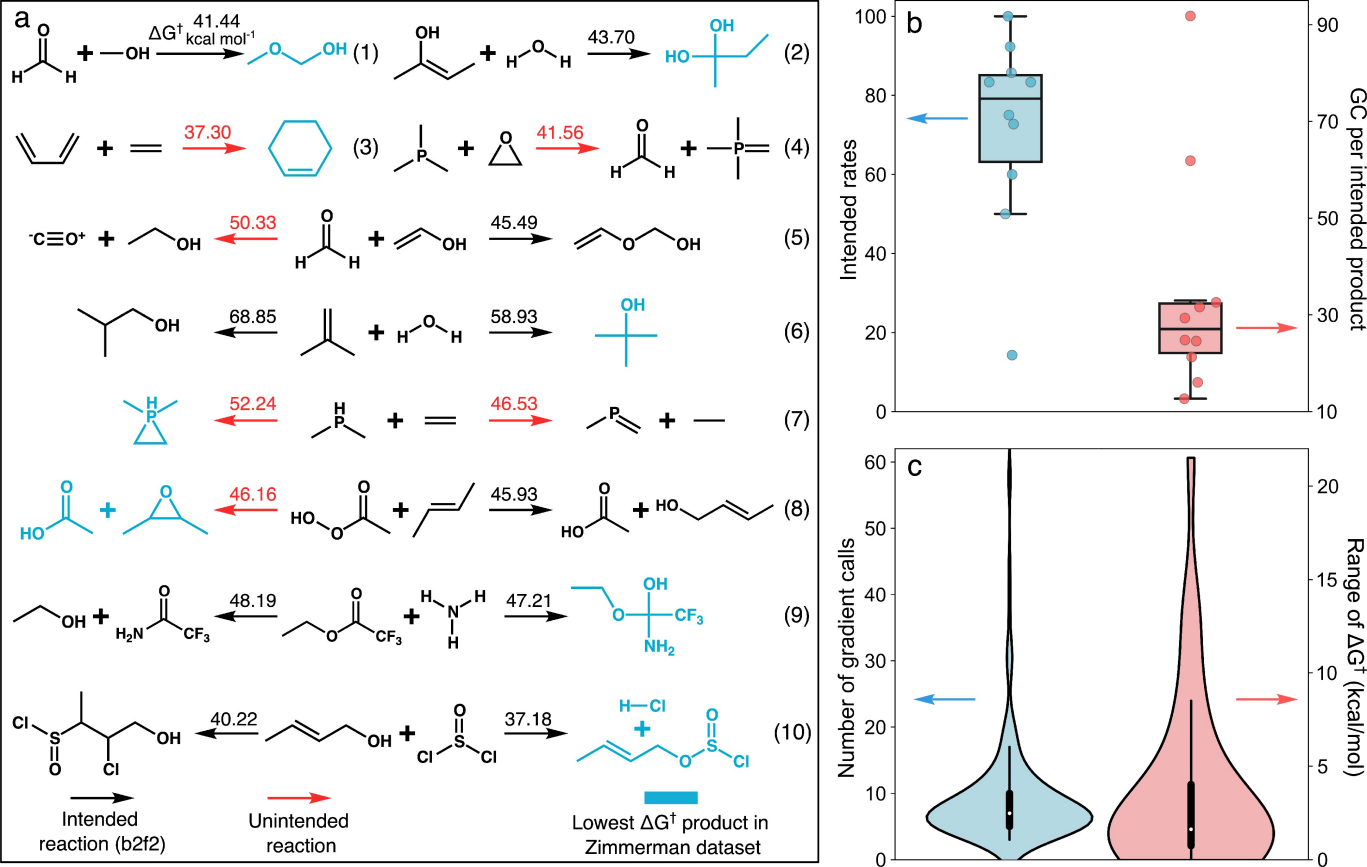
Overview of bimolecular reaction exploration results. a) The minimum activation energy pathways (computed at *ω*B97XD/def2‐TZVP level of theory) for each pair of bimolecular reactants. For the systems where YARP discovers multiple kinetically favorable reactions (i.e. $\Delta G^{†}$ deviation within 5 kcal mol^−1^), both reaction pathways are shown. The lowest barrier pathways reported by Zimmerman are shown in blue, intended (b2f2) reactions are shown in black, and unintended (b3f3, b2f3, etc) are shown in red. b) The intended rates of unique reactions involving each reactant (left axis) and the DFT gradient calls required to discover unique intended products for each reactant (right axis) (c) The distribution of the number of DFT gradient calls required to localize all TSs (left axis) and the distribution of the range of $\Delta G^{†}$ among different reaction conformations associated with each reaction (right axis).

Figure 5a compares the low barrier algorithmically generated pathways with the established reactions. Reactions leading to all established products were discovered during the exploration. Lower barrier reactions were also discovered for reactants 4, 5, 7, and 8 compared with the previously reported reactions. For systems 4 and 5 the previously reported reactions are not even shown in the figure because they are not within 5 kcal mol^−1^ of the lowest barrier reaction. The inclusion of unintended TSs that still correspond to the named reactant leads to positive results in several cases. For example, the Diels‐Alder reaction (system 3) and formation of 2,3‐dimethyloxirane (system 8) are both low barrier b3f3 reactions that are nevertheless discovered via unintended TSs that included the intended reactant in the IRC while exploring b2f2 reactions. Similarly, for system 7 both the expected product (the leftward b1f2 reaction) and an even lower barrier alternative (the right b2f3 reaction) were discovered via unintended transition states. For four out of six of these unintended reactions, other initial conformations led to intended reactions. In these cases, the RMSDs between the unintended and intended TSs are large (*>*1Å), suggesting that the PES is quite distorted by the low barrier channel. This large distortion is also evident from the fact that most of the unintended TSs correspond to endothermic reactions and so are expected to be relatively far from the reactant configuration.

The same four performance statistics are reported for this benchmark (Figure 5b, c). The intended rate varies from 14.3 % to 100 % with an average of 68.0 %. System 4 is an outlier with an intended rate of only 14.3 % (2 over 7), while the intended rate for all other cases are higher than 50 %. However, only 7 products were enumerated for this system, which is insufficient to accurately gauge performance. Additionally, for polyvalent elements, such as phosphorus and sulphur, the elementary reaction step is no longer merely b2f2 and unintended reactions play an essential role. The average DFT level GCs and GCPI of bimolecular reactions are 9.2 and 30.9, respectively, which is sightly larger than the unimolecular study. The number of GCs is correlated with the system size. The higher number of GCs is mainly caused by system 10 which contains 9 heavy atoms, including a sulphur. Excluding this case, the overall cost of the bimolecular benchmark is consistent with the unimolecular systems.

Conformational sampling has a smaller impact on the distribution of $\Delta G^{†}$ for this benchmark compared with the unimolecular benchmarks (Figure 5c). Out of 87 intended reactions, 37 contain more than one intended TS and have an average $\Delta G^{†}$ difference of 3.7 kcal mol^−1^. Only 1 out of 37 reactions has a $\Delta G^{†}$ difference larger than 15 kcal mol^−1^ (this outlier is analyzed in Supporting Information section 7).

### Single‐Step L‐Glucose Pyrolysis Exploration

As a final exploration we have included a study of the single‐step competing pathways for the pyrolysis of L‐glucose. Elucidating the reaction network associated with glucose pyrolysis out to terminal products is a long‐standing and ongoing research topic that goes beyond the present scope. However, the initial pyrolysis step is amenable to algorithmic exploration and glucose represents a significantly larger system than is typically studied using automated exploration algorithms.

**Figure 6:**
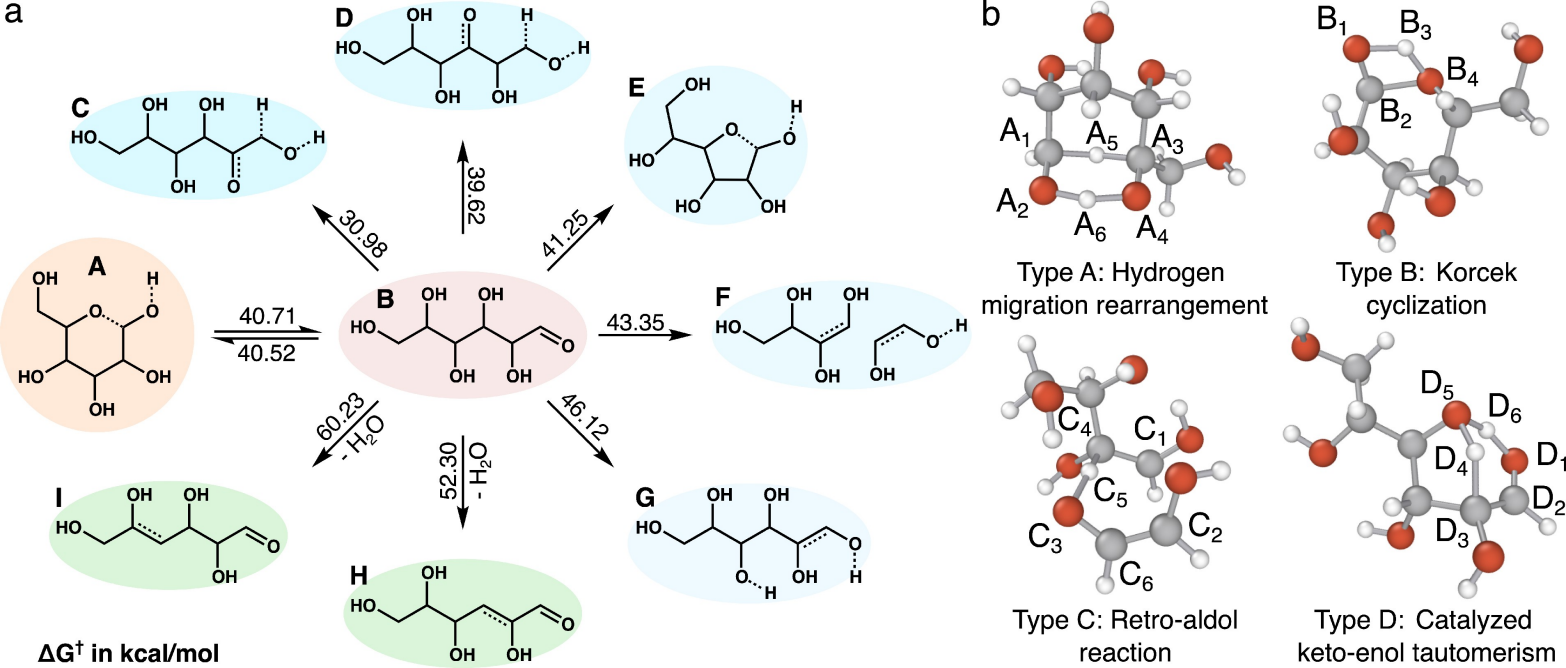
Results from single‐step exploration of L‐glucose pyrolysis reactions. a) Summary of low barrier pathways (computed at B3LYP‐D3/TZVP level of theory) and products discovered by the exploration. β‐D‐glucose (orange) is an L‐glucose precursor. The five discovered intermediates shown in blue have also previously been proposed as leading to specific pyrolysis products. Two water elimination pathways are shown in green that have been proposed in the literature as the initial step of glucose conversion. b) Transition states and reaction mechanisms of four low barrier reaction types, namely hydrogen migration rearrangement, korcek cyclization, retro‐aldol and hydroxyl group catalyzed keto‐enol tautomerism reaction.

For this system, both the b2f2 and cb3f3 elementary reaction steps (see Supporting Information section 1 for detailed description) were used, resulting in 264 and 103 distinct enumerated products, respectively. 167 of these products satisfied a $\Delta H_{r}$ threshold of 80 kJ mol^−1^ and were used for low‐level TS localization and characterizations. Reactions with $\Delta G^{†}<$ 50 kcal mol^−1^ were downselected for high‐level characterization (See Supporting Information section 4 for additional discussion of $\Delta G^{†}$ filtering). After filtering and downselecting, 84 reactions were characterized at the B3LYP‐D3/TZVP level, requiring 1355 DFT level gradient calls and achieving an intended rate of 33.3 % (28/84). Out of the 367 distinct channels that were algorithmically explored, the six lowest barrier products include all previously proposed single‐step intermediates of L‐glucose conversion and one new potentially relevant pathway (Figure 6). Other products are provided in Figure S10.

Starting from L‐glucose, six reactions with $\Delta G^{†}$ lower than 46.12 kcal mol^−1^ (i.e. 2 eV) were discovered. β‐D‐glucose (product A in Figure 6a), the L‐glucose precursor, was discovered with backward (A to B) and forward (B to A) $\Delta G^{†}$ of 40.71 and 40.52 kcal mol^−1^, respectively. The other low barrier reactions produce D‐(−)‐fructose (product C), 3‐hexulose (product D), hexofuranose (product E), 1,2‐ethenediol (product F) and hex‐1‐ene‐1,2,3,4,5,6‐hexaol (product G) with $\Delta G^{†}$ varying from 30.98 to 46.12 kcal mol^−1^. Among these, D‐(−)‐fructose and hex‐1‐ene‐1,2,3,4,5,6‐hexaol formation have been proposed as the first intermediate of various L‐glucose to 5‐hydroxymethylfurfural (HMF) conversion mechanisms.,[^11^, ^39^, ^40^] while 3‐hexulose formation was proposed as the first step of converting L‐glucose to furfural (FF) and hydroxyacetaldehyde (HAA).^[11]^ Two other water elimination reactions (product H and I) that have been proposed as starting points for L‐glucose to 5‐hydroxymethylfurfural (HMF) conversion[^41^, ^42^] were also discovered by the algorithmic exploration. Direct β‐elimination pathways were also discovered during the exploration but exhibited relatively high barriers. In addition to rediscovering all the proposed key first‐step intermediates of L‐glucose conversion, a pathway producing hexofuranose ($\Delta G^{†}=$ 41.25 kcal mol^−1^) as an intermediate, which may have potential relevance for interpreting L‐glucose pyrolysis.

The six low barrier reactions were further classified into four reaction types. The reactions to C and D are hydrogen migration rearrangements (Figure 6b). Two hydrogen atoms A_5_ and A_6_ transfer from carbon atom A_1_ to A_3_ and oxygen atom A_2_ to A_4_, respectively. The TS contains a stable six‐membered ring that explains the low barrier. The reactions from B to A and B to E are actually Korcek cyclizations that also play an essential role in KHP decomposition. The reaction from B to F is a traditional retro‐aldol reaction where a carbon‐carbon bond (C_1_ to C_2_) breaks along with a hydrogen atom transfer (C_5_) from carbon atom C_4_ to C_3_. The reaction producing product G is similar to a keto‐enol interconversion except that the oxygen atom D_5_ serves as a proton shuttle for hydrogen transfer with hydrogen atom D_4_ transferring from D_3_ to D_5_ and hydrogen atom D_6_ transferring from D_5_ to D_1_. This intramolecularly catalyzed mechanism dramatically decreases the activation energy and makes hex‐1‐ene‐1,2,3,4,5,6‐hexaol a kinetically accessible intermediate. With this example, YARP v2.0 shows its capability of maintaining large reaction coverage and low computational cost while describing complex systems.

## Conclusion

The presented benchmarks demonstrate the viability of contemporary reaction exploration algorithms like YARP v2.0 for general‐purpose use on organic reactants. For the diverse range of systems explored, algorithmic exploration discovered all established reaction channels where such comparisons were possible. For all networks and reactants, algorithmic exploration also found new kinetically competitive reaction channels. Moreover, these explorations did not require unusual computational resources and represent a four‐fold reduction in computational cost compared with the previous version of YARP. Despite these ultra‐low costs, YARP v2.0 still incorporates conformational sampling which proved to be important in all benchmarks. Both the resulting reaction barriers and whether the TS was intended or unintended depended strongly on the choice of conformer. This factor will only become more important as automated algorithms begin addressing larger systems with increased conformational complexity. Conformational uncertainty is already a major issue in ongoing TS data generation efforts, where high‐level calculations are being performed on what are often kinetically irrelevant TSs. In total, three unimolecular reaction networks, single‐step reaction channels for ten bimolecular reactants, and one‐step pyrolysis pathways of L‐glucose were reported here, covering C,H,O,N,S,P elements and up to 12 heavy atoms. To the best of our knowledge, this represents the largest TS‐driven exploration benchmark that includes a scheme for conformational sampling. All 581 reactions, including distinct reaction conformations, are provided in the associated data files for use in reactivity prediction and activation energy estimation by interested parties.

Some limitations are also evident from these benchmarks that should encourage further work within the field. First, in the network exploration benchmarks ions and radicals were neglected from further exploration due to their distinct ERSs. Likewise, hypervalent elements showed distinct reactivity relationships that were only discovered via unintended channels. Benchmarking on these species and transition metals, which were also excluded here, are thus obvious extensions of this work. Second, the field would benefit from more diverse low‐level models for estimating reactivity. GFN2‐xTB, which was used here, is quite robust for screening reactions and estimating TSs, but it still shows qualitatively inaccurate behavior for some reactions. We anticipate that recently developed ML‐based potentials may achieve higher accuracy and provide additional low‐cost exploration options. Third, no attempt has been made to consider excited states that may contribute to the calculated barriers and affect the reaction mechanisms associated with non‐adiabatic reactions. To address this, reaction exploration algorithms will benefit from ongoing research towards black‐box multi‐reference methods. Fourth, no attempt was made to use reaction templates to expedite the TS searches. Historically such templates have only been available for an extremely narrow range of chemistries, but with burgeoning reaction data this situation may change in the near‐term and unbiased template‐based approaches might become viable. With these and other foreseeable improvements, black‐box reaction exploration is likely to become a standardized simulation tool available to the chemical community.

## Conflict of interest

The authors declare no conflict of interest.

1

## Supporting information

As a service to our authors and readers, this journal provides supporting information supplied by the authors. Such materials are peer reviewed and may be re‐organized for online delivery, but are not copy‐edited or typeset. Technical support issues arising from supporting information (other than missing files) should be addressed to the authors.

Supporting Information

Supporting Information

Supporting Information

Supporting Information

Supporting Information

## Data Availability

The data that support the findings of this study are available in the Supporting Information of this article. The version of YARP used to perform this study is available through GitHub under the GNU GPL‐3.0 License [https://github.com/zhaoqy1996/YARP/tree/main/version2.0]. Further raw data sources generated by this work are available at https://doi.org/10.6084/m9.figshare.14766624, including raw output files and molecular (reactants, products and transition states) geometries.
