# Supplementary material for: Algorithmic Explorations of Unimolecular and Bimolecular Reaction Spaces
Source: Angew Chem Int Ed Engl. 2022 Oct 17;61(46):e202210693. doi: 10.1002/anie.202210693 (PMC9827825; doi:10.1002/anie.202210693)
Supplement: Supplementary file 1 — Supporting Information [file ANIE-61-0-s004.pdf]

Angewandte Chemie
Eine Zeitschrift der Gesellschaft Deutscher Chemiker

Supporting Information

Algorithmic Explorations of Unimolecular and Bimolecular Reaction Spaces

*Q. Zhao, B. M. Savoie**

Supporting Information

Contents

1 **Methods** **ii**

1.1 Elementary Reaction Enumeration ii

1.2 Pre-pruning the potential products iii

1.3 Geometry Initialization iv

1.4 Transition State Localization v

1.5 Computational Details vii

2 **Activation Energies Calculated at Different Levels of Theory** **viii**

3 **Comparing the First step of KHP Degradation with YARP v1.0 and YARP v2.0** **x**

4 **Reaction Filtering Examples** **xii**

5 **Using DFT as a Low-Level Method with YARP** xiii

6 **Comparing b2f2 and b3f3 Product Enumeration** xiv

7 **Analysis of Outliers** xvi

8 **Machine Learning Classifier for Intended/Unintended TSs** xix

9 **Additional Figures and Tables** xxii

1 Methods

1.1 Elementary Reaction Enumeration

Graph-based ERSs are used by YARP to enumerate potential products for a given set of reactants as a prerequisite for TS searches. These ERSs take the form of rules for "break m bonds" (bm), "form n bonds" (fn), and "transfer x electrons", akin to standard arrow pushing. The differences between the Lewis structures of any reactant and product are expressible in terms of such an ERS (e.g., an S_N2 reaction is a b1f1 reaction). Graph-based ERSs can be considered a generalization of typical named reactions, since the latter are a constrained subset of the former (e.g., a Diels-Alder reaction is a specific occurrence of a b3f3 reaction).

To develop the current benchmarks, we make use of the fact that graph-based ERSs define reaction subspaces that can be comprehensively characterized for relatively large systems. For example, applying all b1f1 reactions to a given nucleophile and molecule would automatically include the set of all possible S_N2 products. This aspect of graph-based ERSs is used here to comprehensively explore the b2f2 reaction subspaces of several neutral closed-shell reactants. For these species, the b2f2 ERS is the simplest reaction rule that yields non-trivial products.[1] As shown below, the TSs of many physically relevant b3f3 reactions have

sufficient configurational overlap with the TSs of b2f2 reactions that they are still discovered during the TS search, even if they are not directly enumerated. For reference, a comparison of the cost and coverage of b2f2 and b3f3 enumeration schemes is provided in Figure S6. For the relatively large l-glucose system, a conditional b3f3 (cb3f3) ERS has also been used that only enumerates b3f3 reactions involving at least one double bond. Empirically we have found that concerted b3f3 reactions are much more likely when one of the broken bonds is a double bond and the number of possible cb3f3 reactions is much less than b3f3 reactions. Illustrations of these three types of ERSs (b2f2, b3f3, cb3f3) are provided in Figure S1.

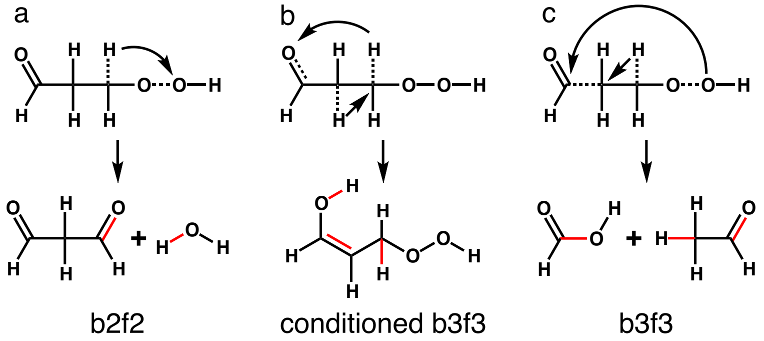

Figure S1: Examples of the three types of elementary reaction steps. (a) b2f2 (b) conditioned b3f3 (c) b3f3

1.2 Pre-pruning the potential products

Even strictly following the simplest b2f2 ERS, the number of possible products increases as $O(N^2)$ where N refers to the number of unique bonds in the reactants. To reduce the search space, the principle of thermodynamic control can be applied to prune the potential products.[2] Since the activation energy is bound from below by the heat of reaction, relatively endothermic reactions may also be safely excluded from a search based on kinetic factors. Within YARP v2.0 an optional threshold on the enthalpies of reaction ($\Delta H_r^{\ominus}$) has been incorporated to down-select reactions before preforming costly TS characterizations (Fig. 1b). Heat of reaction calculations were performed using TCIT,[3,4] an inexpensive and extensible component theory parameterized exclusively from quantum chemistry data.

Finding an optimal value of the $\Delta H_r^{\ominus}$ threshold is possible, but typically impractical. For example, this threshold could be determined in an iterative manner by incrementally raising it and characterizing the TSs (*vide infra*) until all discovered reactions had barriers less than or equal to the threshold, thus exhausting all kinetically relevant products. In practice, setting the threshold this high excludes essentially no channels making the filter useless. Here, the $\Delta H_r^{\ominus}$ threshold was set to be 80 kJ/mol for unimolecular degradation network problems. This value was selected based on comparisons with unfiltered results for the KHP benchmark (Fig. S3) and the observation of no loss of kinetically relevant channels. At room temperature, 80 kJ/mol endothermic products would have sub parts per trillion equilibrium concentrations, but the possibility of their kinetic relevance as intermediates cannot be ruled out *a priori*. The relevant threshold value obviously depends on the reactant. For a very stable reactant, like methyl butanoate (MB), there are relatively few exothermic single-step reactions and thus a higher threshold is appropriate. For the bimolecular reactants and MB a much higher $\Delta H_r^{\ominus}$ threshold of 200 kJ/mol was used to avoid throwing out potentially relevant reactions. For the second step of exploring the MB network, the lower 80 kJ/mol threshold was reused due to the larger number of exothermic single-step reactions after the initial endothermic step.

1.3 Geometry Initialization

The reactions generated by graph-based ERS enumeration must be converted to geometries as a prerequisite for the double-ended TS search. The manner in which this is done is extremely consequential for localizing meaningful TSs, especially for molecules with significant conformational freedom.[5] YARP v2.0 supports two algorithms for generating reaction geometries: the joint-optimization protocol published in the original YARP paper[1] and the conformational sampling plus joint-optimization algorithm that was recently developed by our group.[5] The latter algorithm uses CREST[6] to generate reactants and/or product conformers that are then jointly-optimized and ranked by a random forest classification model

(See Reference 5 for additional details). Both algorithms treat unimolecular and bimolecular reactions on an equal footing. The only distinction is that when using the CREST-based algorithm, multimolecular conformers were discarded if they exhibited a centroid−centroid intermolecular separation greater than twice the sum of the molecular radii. Based on previous benchmarks, these algorithms provide high quality reactive configurations for both unimolecular and bimolecular systems in an automated fashion. Here, both schemes are retained to generate at most $1 + N$ reaction conformations for each attempted reaction (one from the joint-optimization and N from reactant conformational sampling). N is taken as 3 in all case studies, and only those reaction conformations that have an intended score greater than 0.5 predicted by the pretrained random forest classifier were retained for double-ended searches.[5]

1.4 Transition State Localization

TSs are characterized in three steps by YARP. First, a double-ended search is performed to estimate the saddle-point. Here, the growing string algorithm is used for this step, as implemented in the pyGSM package maintained by the Zimmerman group.[7] The highest energy node from the GSM search is used as an initial guess for the TS geometry. Second, a TS refinement is performed starting from the initial guess obtained from the double-ended search. Here Berney optimization[8] is used for this step, as implemented in Gaussian 16.[9] Third, the refined TS is characterized by an intrinsic reaction coordinate (IRC) calculation. The IRC calculation consists of performing gradient descent optimizations along the imaginary frequency mode of the TS, such that the reactant and product corresponding to the TS can be identified. Those TSs where the IRC structures match the enumerated reactant and product (i.e., those that were used to initiate the double-ended search) are referred to as “intended”, otherwise the TS is labeled as “untintended”.

YARP v2.0 introduces two improvements in how these steps are performed and how data are utilized. The first improvement is that the TS localization steps are entirely performed

at a low-level of theory before down-selecting the reactions that merit high-level characterization (Fig. 1c). The low-level characterizations are used to eliminate TSs that are degenerate (e.g., from different reaction conformations) or whose IRC does not include the nominal reactant. In other words, if a TS is unintended but still matches with the reactant, it is retained for high-level TS characterization. The rationale for this is that an unintended TS involving the reactant is still connected to the reaction network that is being explored and therefore of possible relevance. This is the second improvement introduced by YARP v2.0, since previously all unintended TSs were discarded during network exploration. For instance, a TS discovered as part of a b2f2 enumerated reaction may actually correspond to a b3f3 TS, even if the b3f3 ERS was not used directly in enumeration. All down-selected TSs are then refined via high-level Berney optimization using the low-level Berney-optimized TS as an initial guess (Fig. 1d). Here, GFN2-xTB[10] was used as the reference low-level theory (ML potentials and low-level DFT functionals are also available options). For the L-Glucose system, B3LYP-D3/TZVP density functional theory (DFT) was used as the high-level method for comparison with previous work. For all other systems, M05-2x/def2-SVP with D3 dispersion correction[11] was used as the high-level method based on its balance of cost and accuracy and recommendation over B3LYP and M06-2X in a recent benchmark.[12] To provide additional data on the effect of functional choice, up to of the ten lowest barrier reactions involving each reactant and intermediate were refined (both geometries and energies) at the range-separated ωB97XD/def2-TZVP[13] level. The deviations between the two functionals are shown in Figure S2, and the more accurate range-separated values are used in the reaction diagrams reported in the main text.

After obtaining the high-level Berney-optimized TSs, there is the question of whether to perform a high-level IRC calculation. The IRC calculation is typically more expensive than the Berney optimization. Since the reactions have already been filtered by the low-level IRC calculation, just adopting the low-level reaction assignments would save computational cost. However, this would risk misclassifications in cases where the high-level TS underwent

a relatively large geometry rearrangement or where the low-level was inaccurate. In the current study we have performed high-level IRC calculations for all of the unimolecular reaction networks and bimolecular benchmarks. To investigate the feasibility of selectively performing high-level IRC calculations, we have used the high-level IRC data from these benchmarks to train an ML-based XGBoost classifier[14] to estimate the probability that a given high-level TS is intended. The performance and training details for this classifier are provided in the SI section 8. As a proof-of-principle, this classifier is used in the L-Glucose study to down-select TSs predicted to be unintended for IRC calculations.

1.5 Computational Details

YARP v2.0 supports both Gaussian 16[9] and Orca[15] as reference quantum chemistry engines for high-level characterization steps. In the present study, DFT calculations were carried out by Gaussian 16. Gaussian and xTB interfaces were added to the original pyGSM package[7] for performing GSM calculations. All low-level GFN2-xTB calculations were performed with the xTB program (version 6.4.0),[10] while the Gaussian-xtb interface was modified from the gau_xtb package.[16] All simulations were run on a 448 node commodity cluster composed of two Rome CPUs (2.0GHz), 128 effective cores, and 256 GB of memory per node. DFT calculations were performed with 32-core parallelization, while all other calculations were performed as bundled single-core jobs.

All activation energies are reported as free energies at room temperature calculated with respect to the most energetically stable reactant conformer. All reactant and TS energies are reported based on the single point energy plus the zero point energy correction and the room temperature thermal contributions to the molecular enthalpy and entropy estimated using a harmonic partition function calculated based on the normal mode frequencies. These quantities correspond to the standard values calculated and reported in Gaussian 16.[9]

2 Activation Energies Calculated at Different Levels of Theory

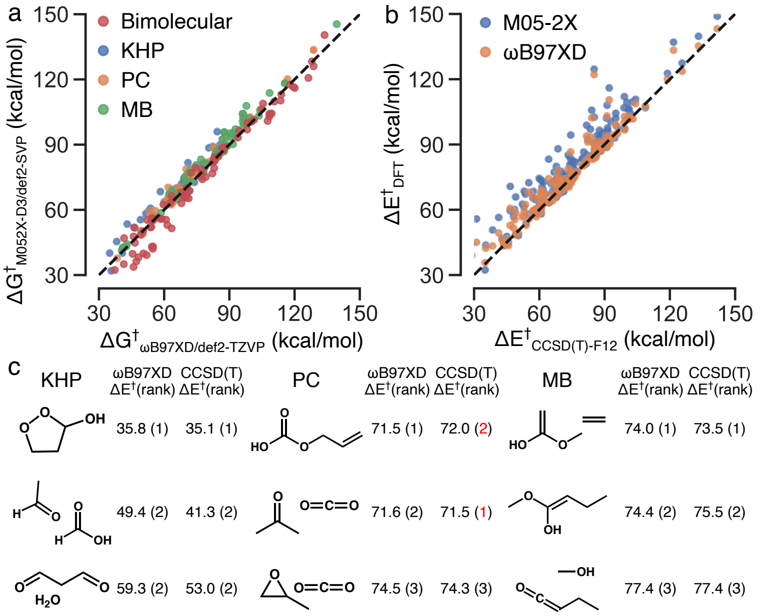

Figure S2: Comparisons between DFT functionals and coupled-cluster results. (a) Correlation plot comparing the activation free energies for the bimolecular and unimolecular systems at the M052X-D3/def2-SVP and ωB97XD/def2-TZVP levels of theory. (b) Correlation plot comparing the activation energies calculated at the M052X-D3/def2-SVP (blue) and ωB97XD/def2-TZVP (orange) with respect to the CCSD(T)-F12/cc-pVDZ-F12 levels of theory. (c) A comparison between the ωB97XD/def2-TZVP and CCSD(T)-F12/cc-pVDZ-F12 activation energies for the three lowest barrier reactions from the first step exploration for KHP, PC, and MD. The ranking of the reactions by their activation energies are shown in parentheses.

The M05-2X functional[17] with D3 dispersion correction[11] and def2-SVP basis set[18] was selected as the high-level method for refining transitions states (TSs) after the low-level GFN2-xTB[10] exploration. To evaluate the consequences of this choice, we selected up to 10 of the most kinetically favorable reactions for each reactant in the KHP, PC, MB networks, and bimolecular benchmarks for re-optimization at the ωB97XD/def2-TZVP[13] level of theory. For the networks, reactions from both the first- and second-layer were included in the comparison. This dataset contains a total of 256 reactions, of which 50, 65, 56, and 85 reactions are from the γ-ketohydroperoxide (KHP), propylene carbonate (PC), methyl butanoate (MB) and bimolecular systems, respectively. The overall mean signed error (MSE)

and mean absolute error (MAE) calculated with respect to the range-separated functional are 1.2 and 3.5 kcal/mol, respectively. The overall intended rate for these reactions is 98% (i.e., only 5 out of 256 reactions become unintended during the Berny optimization at the ωB97XD/def2-TZVP level of theory when starting from the M052x-D3/def2-SVP TSs), which indicates the similarity between the M052x-D3/def2-SVP and ωB97XD/def2-TZVP level potential energy surfaces. These similarities suggest that there is marginal benefit for using the range-separated functional in the current cases. For example, the differences in activation energy between the functionals is typically much smaller than the range of activation energies observed for distinct conformers. Since conformational sampling is neglected in many automated exploration studies, we note that spending time on higher level functionals or multireference calculations without conformational sampling misrepresents the potential sources of error affecting the discovered TSs.

To estimate the errors associated with using DFT rather than a wavefunction based method, the DFT activation energies were also compared with CCSD(T)-F12/cc-pVDZ-F12 (Fig. S2b).[19,20] Due to the relatively high cost and poor scaling of CCSD(T), only the reactions in the KHP, PC, and MB networks (no more than seven heavy atoms) were included in the comparison. The CCSD(T)-F12/cc-pVDZ-F12 single-point calculations were performed on the geometries optimized at the ωB97XD/def2-TZVP level (Fig. S21b). The MSE/MAE of M052x and ωB97XD are 5.7/6.2 and 2.6/3.3 kcal/mol, respectively, compared with CCSD(T)-F12. Both functionals systematically overestimate the barrier heights compared with CCSD(T)-F12/cc-pVDZ-F12. Nevertheless, ωB97XD/def2-TZVP provides accurate kinetic ranking for the investigated systems. For example, the three products with the lowest activation energies of first step of KHP, PC, and MB exploration obtained by ωB97XD/def2-TZVP and CCSD(T)-F12/cc-pVDZ-F12 calculations are summarized in Figure S2c. The top three products and their kinetic rankings predicted by ωB97XD and CCSD(T)-F12 match, with the minor exception of two reactions with nearly identical barriers (PC decomposition examples shown in red).

3 Comparing the First step of KHP Degradation with YARP v1.0 and YARP v2.0

To establish the effects of the down-selection procedures introduced in YARP v2.0 (i.e., the heat of reaction filter and treatment of unintended channels discussed in the main text), we have compared differences with YARP v1.0 in the products that were discovered in the first step of KHP reaction exploration (Fig. S3). In total, YARP v2.0 excluded four intended products that were discovered by YARP v1.0, while discovering four new intended products and seven unintended products. The bottom three missing products were discarded after the reaction enumeration step since they exceed the enthalpy of reaction threshold (80 kJ/mol, around 20 kcal/mol). Based on the YARP v1.0 results, they correspond to kinetically irrelevant reactions, with activation energies more than 40 kcal/mol larger than the most kinetically favorable reaction. The TS search was performed for the first missing product but failed to locate an intended TS with YARP v2.0. This is caused by the additional GFN2-xTB level Berney optimization that is performed in YARP v2.0 (i.e., in v1.0 only the growing-string calculation was performed at the low-level). Nevertheless, this missing product also connected by a reaction with a barrier 40 kcal/mol larger than the most kinetically favorable reaction.

On the other hand, four newly discovered products indicate that YARP v2.0 increased the intended reaction coverage. This is mainly due to conformational sampling in YARP v2.0, albeit in this case the resulting intended products are all relatively high barrier. Many unintended channels were also discovered, including two of potential kinetic relevance (Fig. S3).

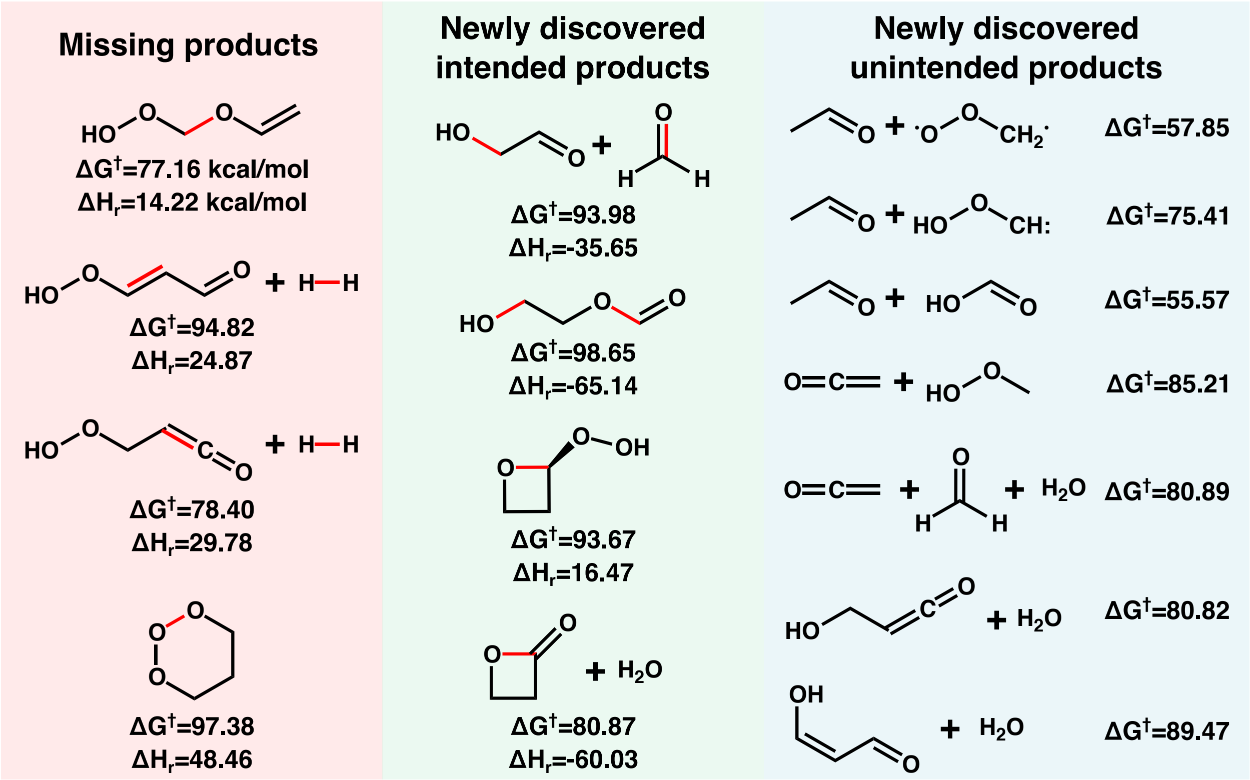

Figure S3: Summary of missing intended products (left), newly discovered intended products (middle) and newly discovered unintended products (right) when exploring the first step degradation pathways of KHP with YARP v2.0 and YARP v1.0. The free energies of activation ($\Delta G^{\dagger}$) of missing and newly discovered reactions are computed at B3LYP/6-31+G* level by YARP v1.0 and M05-2x/def2-SVP with D3 dispersion correction by YARP v2.0, respectively. The enthalpy of reaction (ΔH_r) of intended reactions are computed by TCIT. The energies are reported in kcal/mol.

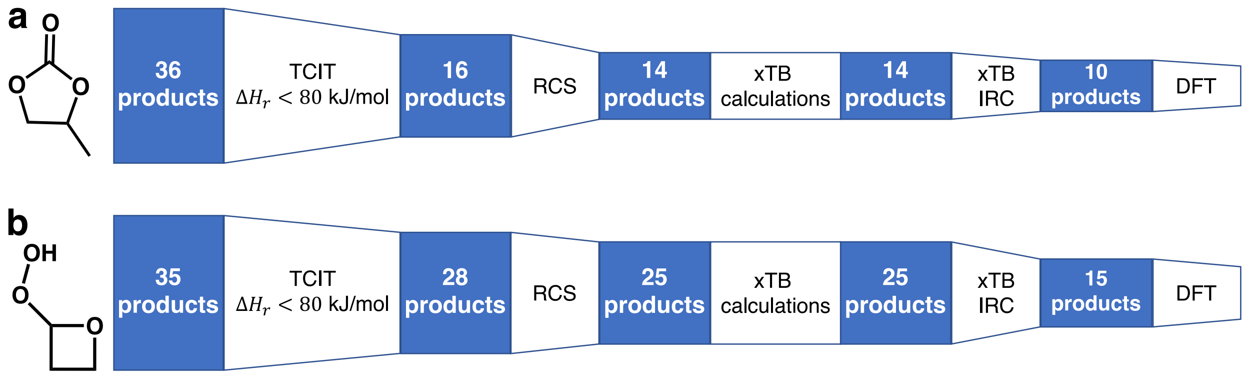

Figure S4: Examples showing how YARP v2.0 down-selects potential reactions before performing high-level calculations. RCS is short for reaction conformational sampling.

4 Reaction Filtering Examples

Two examples are provided to illustrate how YARP v2.0 down-selects the potential reactions and reaction conformations (Fig. S4). The first example, propylene carbonate (PC), is the reactant with the highest down-selection rate among reactants with more than 5 DFT level products. The second example, oxetane-2-yl hydroperoxide (OH), is an intermediate in the KHP network that exhibits a near-average down-selection rate.

In the first step, TCIT[3,4] is applied to predict the enthalpy of formation (ΔH_{f}) of the reactant and the enumerated b2f2 products. With the enthalpy of reaction ($\Delta H_r^{\ominus}$) threshold of 80 kJ/mol, 20 and 7 products are excluded in the PC and OH systems, respectively. Reaction conformational sampling (RCS) is then applied to the remaining products. In this step, 2 and 3 products are excluded for PC and OH, respectively, because no reaction conformations were generated with sufficiently high intended probability for these products. In the third step, GFN2-xTB level growing string method (GSM) and Berny TS optimization are performed and the products with no successful reaction channel (i.e. either GSM or TS optimization do not converge) are excluded. In both systems, all products pass this step. At this stage if reaction conformations result in a same TS (i.e. showing a single point energy difference smaller than 10^{-4} Hartree) they will be treated as duplicates and only one will be kept. The last down-selection step is the GFN2-xTB level intrinsic reaction coordinate (IRC) calculation. If the low-level IRC analysis is unintended and the reactant is not included in either of the IRC endpoints then the reaction will be excluded from high-level characterization. For PC and OH, 4 and 10 products, respectively, were excluded since none of their IRCs satisfied the retention criteria. Note that since multiple reaction conformations are used there are possibly multiple IRCs associated with a product. In total, this down-selection process excludes 72.2% and 57.1% enumerated products from high-level characterization for PC and OH, respectively, which helps to dramatically decrease the computational cost. Section 5 discusses whether this process leaves out important reactions

due to the inaccuracy of the GFN2-xTB calculations.

Activation energy threshold. Besides the above mentioned down-selection procedures, an activation energy threshold ($\Delta G^{\dagger}_{\mathrm{thresh}}$) can be set to avoid exploring the reactions with relatively high barriers. For example, when studying reaction conditions at 100°C, only reactions with $\Delta G^{\dagger} \leq 45$ kcal/mol exhibit appreciable kinetics on lab timescales.[21] In YARP this filter is applied after the low-level TS calculation and for screening high-level unintended TSs. A 5 kcal/mol energy window is added when applying the low-level filter to acknowledge the relative inaccuracy of the low-level method (GFN2-xTB, in this case). Thus, setting a $\Delta G^{\dagger}_{\mathrm{thresh}}$ of 45 kcal/mol will exclude reactions with $\Delta G^{\dagger} > 50$ kcal/mol computed at GFN2-xTB level and unintended reactions with $\Delta G^{\dagger} > 45$ kcal/mol computed at the DFT level. This filter was only used in the present study for the L-glucose pyrolysis benchmark.

5 Using DFT as a Low-Level Method with YARP

Table S1: Statistics of additional test with B3LYP/6-31+G* as low cost method. The "reactions attempted" column corresponds to the number of TSs that were down-selected for eventual high-level characterization in each scenario. The "I/U" column reports the number of intended and retained unintended products found after the high-level IRC step.

Reactant	Input products	xTB high-level reactions attempted	xTB I/U	B3LYP high-level reactions attempted	B3LYP I/U
O[C@H]1CCOO1	23	11	5/4	10	3/4
C1COOC1=O	7	1	1/0	2	1/1
C1COC1=O	6	2	2/0	2	2/0
O=C1OCCCO1	9	4	4/0	7	5/2
O=c1occo1	1	0	0/0	0	0/0
CP(C)C.O1CC1	7	7	1/2	6	2/2

To investigate GFN2-xTB down-selection as a potential source of error, six reactants with large low-level down-selection rate (step 3 and 4 in the process illustrated in Fig. S4) were reevaluated using B3LYP/6-31+G* as a low-level method (Table S1). The keyword "I/U" refers to the number of intended and retained unintended products and "xTB" ("B3LYP") refers to the YARP v2.0 results using xTB (B3LYP/6-31G*) as the low-level method. For

four out of the six systems, using xTB as the low-level method results in finding the same (or more) intended channels as using B3LYP/6-31+G*. For 1,3-dioxan-2-one (O=C1OCCCO1) and trimethylphosphane plus oxirane (CP(C)C.O1CC1), using xTB as a low-level method misses one intended channel. However, these missed reactions are relatively high activation energy and are not considered important. Using B3LYP/6-31+G* as a low-level method does result in two retained unintended products, both from the PC network, that are more kinetically favorable than the reactions explored by xTB for these two reactants (Fig. S5).

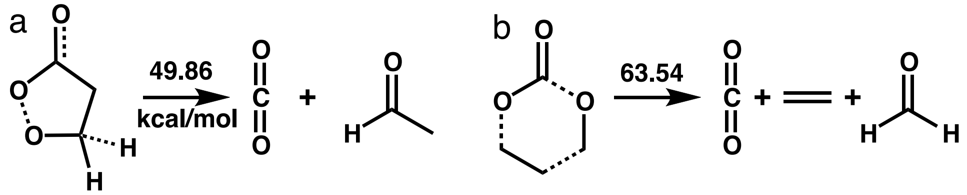

Figure S5: Two kinetically favorable reactions that are discovered as retained unintended channels using B3LYP/6-31+G* as a low-level method that are missed when using xTB.

Although the two methods perform similarly as low-level chemistries, they exhibit dramatic differences in computational cost. The walltime (single core) of xTB level exploration (run as bundled 1 CPU jobs) is 32533.97s (GSM:22495.37s, TS optimization:3313.00s, IRC:6725.60s) while the walltime of B3LYP/6-31+G* level exploration (run as bundled 4 CPU jobs) is 2922592.68s (GSM:1894650.68s, TS optimization:657659.20s, IRC:370282.80s). So the computational cost of B3LYP/6-31+G* level of theory is around 90 times the cost of xTB, which roughly matches with our previous observations for YARP v1.0.[1]

6 Comparing b2f2 and b3f3 Product Enumeration

The selection of the elementary reaction step (ERS) is essential in YARP, since it determines the reaction coverage and computational cost. This topic was discussed extensively in the original YARP publication, in particular as regards under what conditions it is necessary to include higher order ERSs like b3f3 for neutral closed-shell reactants.[1] In that study we found that remarkably little chemistry was missed when excluding b3f3, but reactions like Diels-

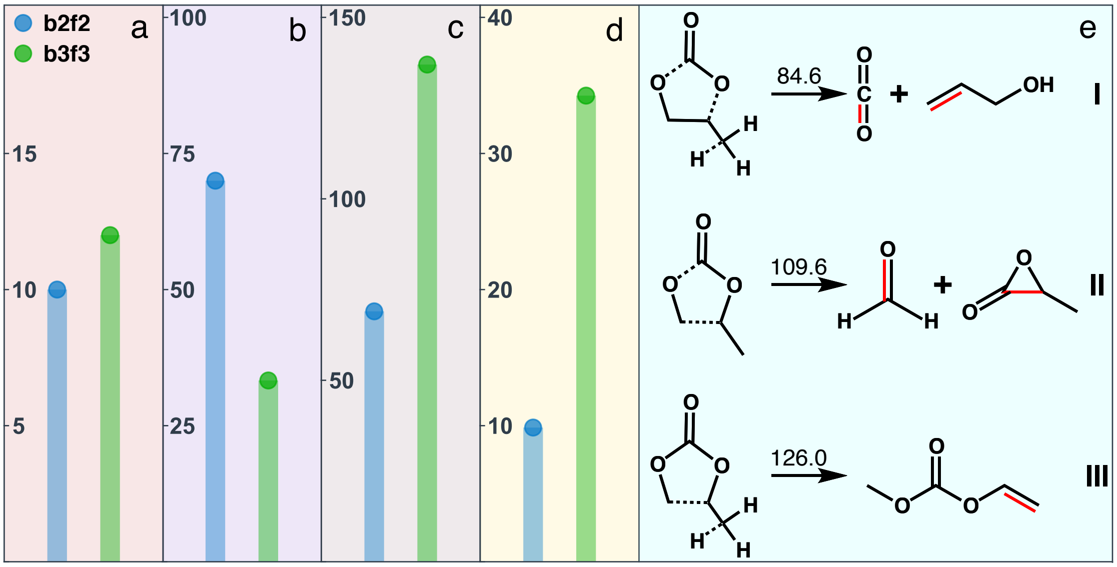

Figure S6: Comparison of b2f2 and b3f3 reaction explorations. a. Number of potential products. b. The intended rates of unique reactions. c. Total number of DFT level gradient calls. d. Number of DFT level gradient calls per intended product. e. Three reactions that were only discovered during b3f3 exploration. Activation energies are shown over each arrow in kcal/mol.

Alder were notable exceptions. Since the inclusion of unintended channels in YARP v2.0 results in the spontaneous discovery of some b3f3 and b4f4 reactivity compared with YARP v1.0, it is worthwhile to revisit the question of what chemistry is missed by excluding the b3f3 ERS in the enumeration step. We remind the reader that the summary observation from our experience with YARP is that b3f3 and b4f4 reactions are much more poorly conditioned for TS convergence (e.g., they often exhibit multiple intervening TSs) while adding a large number of additional products to attempt TS searches for. The net result is very low success and intended rates, which motivates the search for alternative to grossly attempting all of these reactions.

To compare the coverage and cost of b2f2 and b3f3 exploration with inclusion of unintended reactions, the first step of reaction exploration for PC was reperformed with the b3f3 ERS and the number of products, intended rate, number of DFT gradient calls (GC) and gradient calls per intended product (GCPI) were compared with only using b2f2 enumeration (Fig. S6). The numbers of b2f2 products (10) and b3f3 products (12) are similar (Fig.

S6a); however, the number of b3f3 products grows much faster than b2f2 products as system sizes increase. The intended rate of b2f2 exploration is 70%, which is over twice the intended rate of b3f3 exploration (33.3%, Fig. S6b). This ratio reflects the difficulty of converging b3f3 TSs (and potentially their relative scarcity on the chemical potential energy surface) compared with b2f2 TSs. As for the computational cost, b2f2 exploration required 69 GCs, while b3f3 required 137 GCs, which is twice the cost of b2f2 exploration alone. The larger number of GCs is indicative of the b3f3 TSs undergoing larger structural changes from the low-level estimation on average compared with b2f2, which is also indirectly related to the low intended b3f3 rate. The GCPI of b3f3 is approximately three times the GCPI of b2f2 exploration, which summarizes the low efficiency of b3f3 searches compared with b2f2.

Despite the additional costs of b3f3, the question remains: what products are missing from the b2f2 exploration that can only be found using b3f3 enumeration? Only one intended reaction (I) and two unintended reactions (II, III) were discovered by b3f3 exploration that were not characterized during b2f2 exploration (Fig. S6e). The two unintended reactions are actually b2f2 reactions that were enumerated by the b2f2 search but were excluded from exploration based on the $\Delta H_r^{\ominus}$ threshold. Thus, only one additional reaction is authentically discovered by b3f3 exploration, and in this case is high barrier and kinetically irrelevant. Besides this testing demonstration, we note that the Diels-Alder reaction, the entire Korcek mechanism, as well as other b1f1,b2f3,b3f3 and even b4f4 reactions are reported in the main text and were discovered as retained unintended channels using only b2f2 enumeration to guide the search. Thus, we recommend b2f2 or conditional b3f3 searches as typically striking a balance between reaction coverage and computational cost for relatively simple organic reaction exploration.

7 Analysis of Outliers

This section discusses the reactions that were outliers with respect to the number of gradient calls and range of $\Delta G^{\dagger}$ from the unimolecular and bimolecular reaction explorations. The

reactions with a number of GC greater than 30 or a range of $\Delta G^\dagger$ larger than 15 kcal/mol were regarded as outliers and were analyzed in detail.

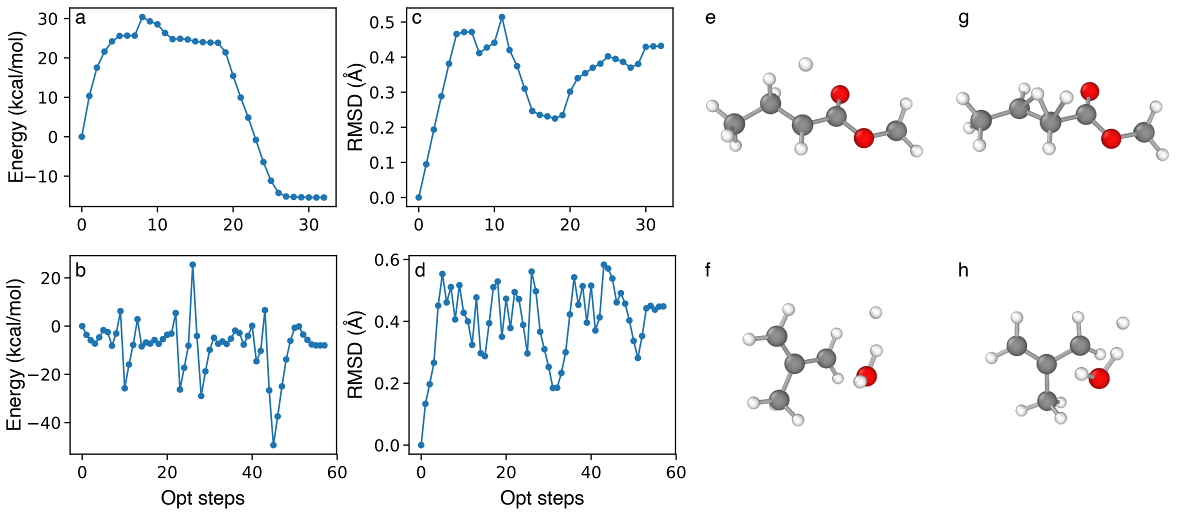

Figure S7: Detailed results for two intended outliers with respect to the number of GCs. (a-b) Energy change during the TS optimization of cases 1 and 2. (c-d) Root-mean-square-displacement (RMSD) change with respect to the starting geometry during the TS optimization of cases 1 and 2. (e-f) Starting geometries (xTB level TSs) of cases 1 and 2. (g-h) Final geometries (DFT level TSs) of cases 1 and 2.

In total 11 transition state optimization tasks took more than 30 gradient calls (GCs) to converge an M052X-D3/def2-SVP level TS starting from a GFN2-xTB level TS initial guess. Among them, only 2 TS were intended after the optimization while the other 9 TS optimizations dramatically changed the TS structures and resulted in unintended TSs. The Berny optimization trajectories of those two cases were analyzed (Fig. S7). The energy and RMSD difference curves illustrate two distinct reasons for the large number of GCs. In the first case, one hydrogen atom undergoes a slow roam towards its final position. In the second case, the optimization procedure gets stuck in an oscillation typically of large changes in the potential compared with the step size. Both cases appear to be artifacts of the Berny optimization and motivate the implementation of more robust and efficient optimization methods in YARP.

In total 12 reactions were found with a $\Delta G^\dagger$ range larger than 15 kcal/mol (Fig. S8), involving 6 unimolecular decomposition reactions, 5 unimolecular transformation reactions

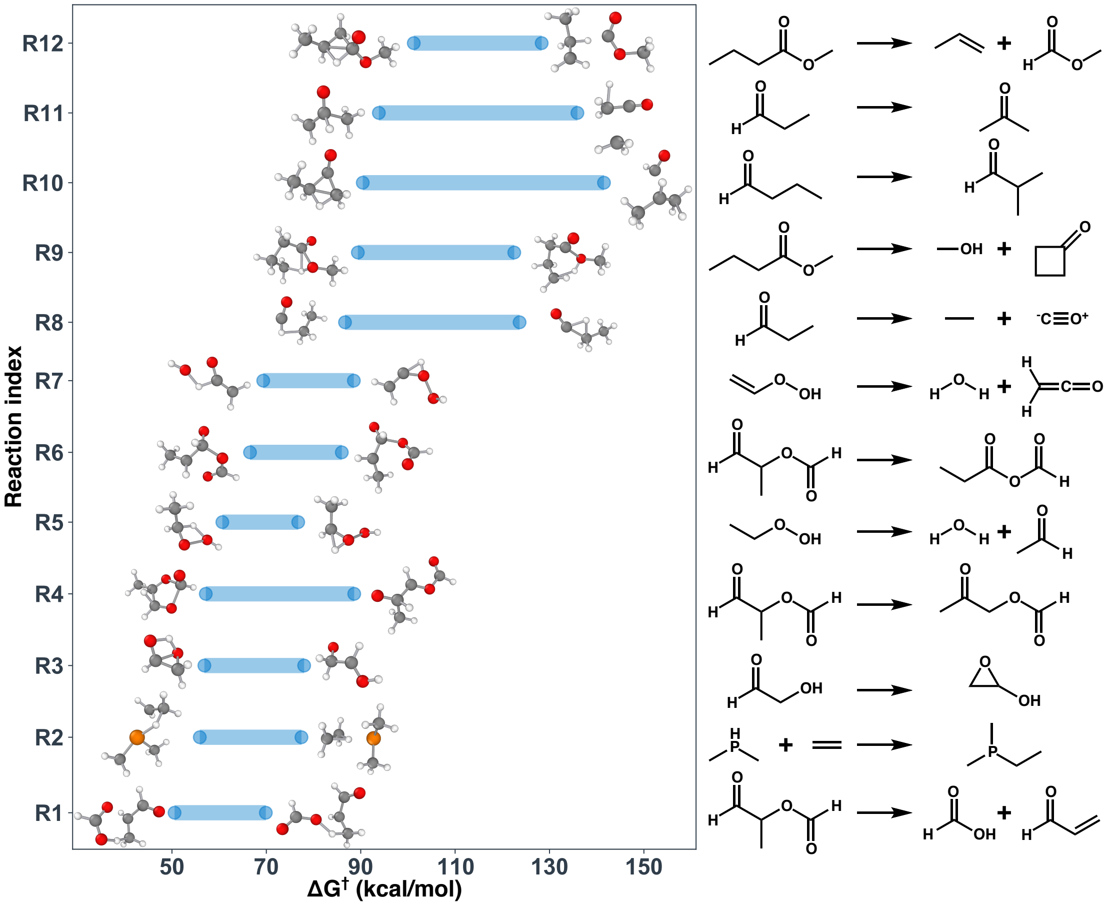

Figure S8: 12 reactions exhibiting a range of activation energies larger than 15 kcal/mol for distinct starting conformations. The geometries of the lowest and highest activation energy transition states are shown (grey: Carbon, red: Oxygen, yellow: Phosphorus, white: Hydrogen).

and 1 bimolecular combination reaction. A common factor in many cases (R1, R4, R5, and R6) is if the lowest energy TS conformation contains a stable ring structure. The TS localized for other conformers is much higher energy if it does not adopt the cyclic structure. Another factor in many cases (R2, R3, R7, R8, R9, R10, R11 and R12) is if a concerted pathway is possible only via certain conformations. For example, the high activation energy TS for many of these cases are bimolecular, whereas the low activation energy pathway involves a unimolecular intramolecular TS.

8 Machine Learning Classifier for Intended/Unintended TSs

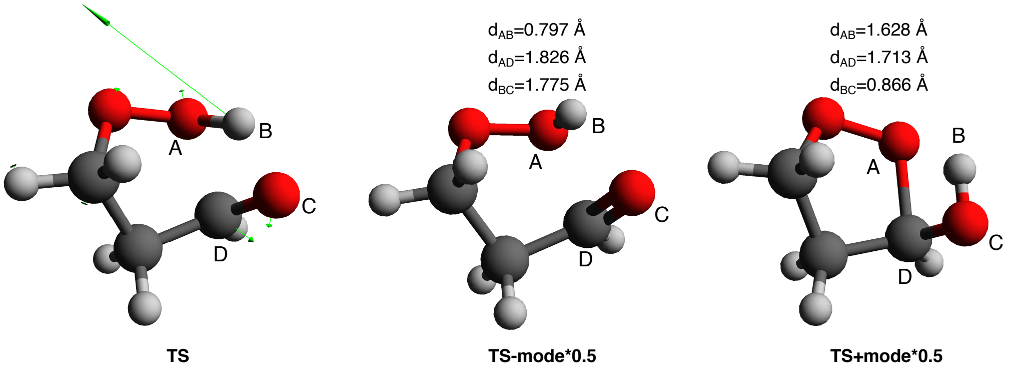

Figure S9: An example of the transition state structure and imaginary frequency mode (left) of the Korcek reaction introduced in the main text; structure of backward (middle) and forward (right) direction.

As described in the methods (Section 2), the IRC calculation is one of the most expensive steps of the TS characterization workflow. Ideally, high-level IRCs would only be performed on unintended reaction channels for the purpose of identifying the end-points associated with the discovered transition state. To investigate the feasibility of screening TSs in advance of the high-level IRC, we have trained an XGBoost classifier to label the high-level TSs using training data from the unimolecular networks and bimolecular reaction benchmarks. As a demonstration of the cost-savings potential of this approach, the resulting model was used in the L-Glucose exploration to down-select unintended TSs for high-level IRC calculations.

We have previously shown that a simple random forest classifier can be effectively trained using general geometric descriptors for down-selecting reaction conformations.[14] Here have followed a similar approach, but have used features based on the imaginary normal mode of the TS (which gives a sense of direction in configuration space with respect to the reactant and product geometries)[22,23] in addition to geometric factors. As a prerequisite to calculating the features, the distance changes (DCs) associated with displacement along the imaginary normal mode were calculated between each pair of nearby atoms. Atoms were defined as near each other if they were within twice their hard shell radii (as estimated from tabulated atomic

radii from the Universal Force Field[24]). The distance change was defined as the difference between the separations of each atom pair after a positive and negative displacement along the imaginary mode (transition state structure plus/minus half of the imaginary frequency mode, Fig. S9). The atomic separations were calculated for all nearby pairs in each displaced geometry, then the difference between the separations in each geometry was calculated as each DC.

Two input features were calculated as the average ($\mathrm{IDC_{mean}}$) and minimum ($\mathrm{IDC_{min}}$) value of the DCs for active bonds (bonds that break/form in the target reaction, denoted as intended distance change, IDC). A third feature was based on the "unexpected" distance changes (UDC) associated with displacement along the imaginary mode, which was calculated from the DCs of inactive bonds (i.e., bonds not undergoing formation or breakage in the reaction) that were larger than $\mathrm{IDC_{min}}$. All UDCs were summed up as $\mathrm{UDC_{sum}}$ to describe the unintended motions with respect to the target reaction. Finally, a geometry-based feature, $\mathrm{S_{max}}$, was calculated as the maximum separation between each active bond in the transition state geometry. In the example of Figure. S9, the separation between atom pairs (A,B), (A,D) and (B,C) were calculated and $\mathrm{S_{max}} = \max\{d_{\mathrm{AB}}, d_{\mathrm{AD}}, d_{\mathrm{BC}}\} = d_{\mathrm{AD}} = 1.770$Å. These four features were used to train a generic XGBoost model (M1) for classifying unintended and intended TSs (*vide infra*). These features are independent of the user's choice of low-level or high-level model chemistry. Thus, M1 does not use any information from the low-level IRC calculation or TS, which are generated by default in YARP v2.0. A comparison between M1 and a model that uses this additional information (M2) is included below.

Four additional features were developed to describe the structural changes occurring between the low-level TS geometry and the high-level TS for use in featurizing the M2 model. The root-mean-squared displacements (RMSD) of the whole structure and of the atoms involved in the reaction (i.e., involved in bonds that break or form) were calculated between the high-level and low-level TSs as two additional features to represent the structural change. The summation of bond length changes between the high-level and low-level TSs

was also included as a feature to highlight potential bond changes. Finally, the low-level IRC classification (intended or unintended) served as an eighth indicator for the M2 model. In contrast to the first four features, the latter four are specific to differences between specific low-level and high-level models and are thus less transferable. As shown below, there is a marginal performance difference between these models.

Table S2: Performance of different models for characterizing the transition states

	precision	recall	f1 score
M1	0.96	0.92	0.94
M2	0.96	0.96	0.96
Grambow	0.91	0.78	0.84

Since the high-level IRC dataset from the unimolecular reaction networks and bimolecular reactions is relatively small (1065 reactions) we experimented with relatively simple and transferable ML models for training the intended/unintended classifier, including logistic regression, decision tree, random forest, and XGBoost. Among them, the XGBoost model obtained the highest precision (not shown) and was selected for training the M1 and M2 models. The whole dataset was composed of 596 intended TSs (true) and 469 unintended TSs (false) and a random 80:20 training:testing split was used. A grid-search and 5-fold cross-validation using the training data was used to determine optimal values for the learning rate (lr), maximum decision tree depth (max_depth), minimum sum of instance weight needed in a child (min_child_weight) and minimum loss reduction required to make a further partition (gamma). After the same tuning procedure, the performance of the M1 and M2 model on the same testing set was provided in Table S2, along with the performance of an earlier model trained by Grambow et al.[22] that solely uses the largest bond length change in the imaginary frequency mode for comparison. The comparison shows both M1 and M2 model perform better than the Grambow model, which misclassifies 22% of intended channels as unintended. Meanwhile, the addition of the latter four indicators results in only a 0.04 increase in "recall", which implies that the general M2 model is sufficient for characterizing

the transition states.

9 Additional Figures and Tables

Table S3: Summary of YARP performance on the 3-hydroperoxypropanal network prediction benchmark.

	Attempted products	DFT-level optimized products	Intended products	Intended product (single conformation)	Success rate	Intended rate
KHP step1	21	19	13	7	100.0%	61.9%
KHP step2	199	154	93	42	94.8%	60.4%
PC step1	14	10	7	4	100.0%	52.6%
PC step2	95	80	63	21	100.0%	78.8%
MB step1	40	38	20	12	100.0%	52.6%
MB step2	141	107	66	32	100.0%	61.7%
bimolecular	136	122	83	38	100.0%	68.0%
Total	678	530	345	156	98.5%	65.1%

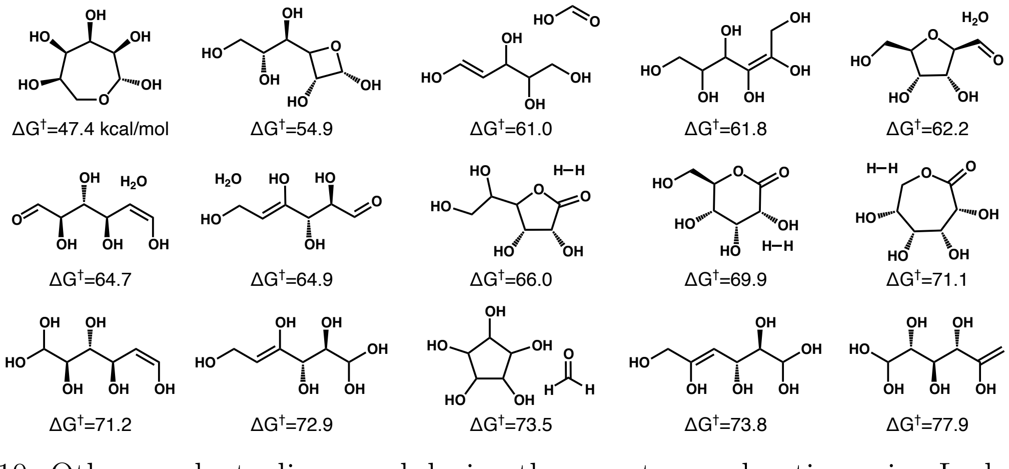

Figure S10: Other products discovered during the one-step exploration using L-glucose as a reactant. These 15 products have higher activation energies than the low-barrier pathways highlighted in the main text.

References

(1) Zhao, Q.; Savoie, B. M. Simultaneously improving reaction coverage and computational cost in automated reaction prediction tasks. *Nat. Comput. Sci.* **2021**, *1*, 479–490.

(2) Suleimanov, Y. V.; Green, W. H. Automated discovery of elementary chemical reaction steps using freezing string and Berny optimization methods. *J. Chem. Theory Comput.* **2015**, *11*, 4248–4259.

(3) Zhao, Q.; Savoie, B. M. Self-Consistent Component Increment Theory for Predicting Enthalpy of Formation. *J. Chem. Inf. Model.* **2020**, *60*, 2199–2207.

(4) Zhao, Q.; Iovanac, N. C.; Savoie, B. M. Transferable Ring Corrections for Predicting Enthalpy of Formation of Cyclic Compounds. *J. Chem. Inf. Model.* **2021**, *61*, 2798—2805.

(5) Zhao, Q.; Hsu, H.-H.; Savoie, B. Conformational Sampling for Transition State Searches on a Computational Budget. *J. Chem. Theory Comput.* **2022**, *18*, 3006–3016.

(6) Pracht, P.; Bohle, F.; Grimme, S. Automated exploration of the low-energy chemical space with fast quantum chemical methods. *Phys. Chem. Chem. Phys.* **2020**, *22*, 7169–7192.

(7) Aldaz, C.; Kammeraad, J. A.; Zimmerman, P. M. Discovery of conical intersection mediated photochemistry with growing string methods. *Phys. Chem. Chem. Phys.* **2018**, *20*, 27394–27405.

(8) Schlegel, H. B. Optimization of equilibrium geometries and transition structures. *J. Comput. Chem.* **1982**, *3*, 214–218.

(9) Frisch, M. J. et al. Gaussian 16 Revision C.01. 2016; Gaussian Inc. Wallingford CT.

(10) Bannwarth, C.; Ehlert, S.; Grimme, S. GFN2-xTB—An accurate and broadly parametrized self-consistent tight-binding quantum chemical method with multipole electrostatics and density-dependent dispersion contributions. *J. Chem. Theory Comput.* **2019**, *15*, 1652–1671.

(11) Grimme, S.; Antony, J.; Ehrlich, S.; Krieg, H. A consistent and accurate ab initio parametrization of density functional dispersion correction (DFT-D) for the 94 elements H-Pu. *J. Chem. Phys.* **2010**, *132*, 154104.

(12) Goerigk, L.; Hansen, A.; Bauer, C.; Ehrlich, S.; Najibi, A.; Grimme, S. A look at the density functional theory zoo with the advanced GMTKN55 database for general main group thermochemistry, kinetics and noncovalent interactions. *Phys. Chem. Chem. Physics* **2017**, *19*, 32184–32215.

(13) Chai, J.-D.; Head-Gordon, M. Long-range corrected hybrid density functionals with damped atom–atom dispersion corrections. *Phys. Chem. Chem. Phys.* **2008**, *10*, 6615–6620.

(14) Chen, T.; Guestrin, C. XGBoost: A Scalable Tree Boosting System. Proceedings of the 22nd ACM SIGKDD International Conference on Knowledge Discovery and Data Mining. 2016; pp 785—794.

(15) Neese, F. The ORCA program system. *Wiley Interdiscip. Rev.: Comput. Mol. Sci.* **2012**, *2*, 73–78.

(16) Lu, T. gau_xtb: A Gaussian interface for xtb code. 2020; `http://sobereva.com/soft/gau_xtb`.

(17) Zhao, Y.; Schultz, N. E.; Truhlar, D. G. Design of density functionals by combining the method of constraint satisfaction with parametrization for thermochemistry, thermochemical kinetics, and noncovalent interactions. *J. Chem. Theory Comput.* **2006**, *2*, 364–382.

(18) Weigend, F.; Ahlrichs, R. Balanced basis sets of split valence, triple zeta valence and quadruple zeta valence quality for H to Rn: Design and assessment of accuracy. *Phys. Chem. Chem. Phys.* **2005**, *7*, 3297–3305.

(19) Knizia, G.; Adler, T. B.; Werner, H.-J. Simplified CCSD (T)-F12 methods: Theory and benchmarks. *J. Chem. Phys.* **2009**, *130*, 054104.

(20) Bischoff, F. A.; Wolfsegger, S.; Tew, D. P.; Klopper, W. Assessment of basis sets for F12 explicitly-correlated molecular electronic-structure methods. *Mol. Phys.* **2009**, *107*, 963–975.

(21) Das, T.; Ghule, S.; Vanka, K. Insights into the origin of life: Did it begin from HCN and H2O? *ACS Cent. Sci.* **2019**, *5*, 1532–1540.

(22) Grambow, C. A.; Pattanaik, L.; Green, W. H. Reactants, products, and transition states of elementary chemical reactions based on quantum chemistry. *Sci. Data* **2020**, *7*, 1–8.

(23) Young, T. A.; Silcock, J. J.; Sterling, A. J.; Duarte, F. autodE: Automated Calculation of Reaction Energy Profiles—Application to Organic and Organometallic Reactions. *Angew. Chem., Int. Ed.* **2021**, *60*, 4266–4274.

(24) Rappé, A. K.; Casewit, C. J.; Colwell, K. S.; Goddard III, W. A.; Skiff, W. M. UFF, a full periodic table force field for molecular mechanics and molecular dynamics simulations. *J. Am. Chem. Soc.* **1992**, *114*, 10024–10035.
